# Design and 3D Printing of Polyacrylonitrile‐Derived Nanostructured Carbon Architectures

**DOI:** 10.1002/smsc.202300275

**Published:** 2024-02-27

**Authors:** Valentin A. Bobrin, Haira G. Hackbarth, Yin Yao, Dipan Kundu, Nicholas M. Bedford, Rhiannon P. Kuchel, Jin Zhang, Nathaniel Corrigan, Cyrille Boyer

**Affiliations:** ^1^ Cluster for Advanced Macromolecular Design School of Chemical Engineering University of New South Wales Sydney NSW 2052 Australia; ^2^ School of Chemical Engineering University of New South Wales Sydney NSW 2052 Australia; ^3^ Electron Microscope Unit Mark Wainwright Analytical Centre University of New South Wales Sydney NSW 2052 Australia; ^4^ School of Mechanical and Manufacturing Engineering University of New South Wales Sydney NSW 2052 Australia; ^5^ Australian Centre for Nanomedicine School of Chemical Engineering University of New South Wales Sydney NSW 2052 Australia

**Keywords:** 3D printing, nanostructured materials, polymerization‐induced microphase separation, self‐assembly

## Abstract

Nanostructured carbon materials with designer geometries are of great interest for a wide range of energy‐based and environmental applications due to their tunable microstructure, which allows for optimized properties and performance, as well as their ability to be shaped in complex three‐dimensional (3D) geometries suited for targeted applications. However, achieving a controllable way for preparing nanostructured carbon materials with precise macroscale control has proven to be challenging. Herein, a straightforward approach for 3D printing of nanostructured polyacrylonitrile (PAN)‐derived carbon materials controlled by employing self‐assembling resins in liquid crystal display printing is presented. The correlation between resin composition, printing parameters, and PAN thermal transformation conditions is identified using a combination of thermoanalytical and structural techniques. The nanostructured PAN materials are readily transformed into carbon with a voided microstructure while retaining the original macro‐architecture of the 3D printed polymer precursor objects. The resulting carbon materials are electrically conductive and feature nitrogen active sites controlled by pyrolysis temperature. This method offers a simple way to produce nanostructured carbon‐based materials with an arbitrary shape, presenting the possibility of advantageous characteristics for a range of potential applications in both the fields of energy and the environment.

## Graphical abstract

This study explores the synthesis of nanostructured carbon materials with customizable geometries for diverse applications in energy and the environment. A novel method for 3D printing polyacrylonitrile‐derived carbon by employing self‐assembling resins in liquid crystal display printing is reported.

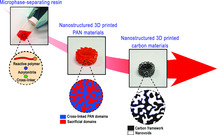

## 1 Introduction

Nanostructured nitrogen (N)‐doped carbon materials have emerged as an important class of materials with a broad range of applications in energy and environment, including their use as supercapacitors,^[^ ^1^ ^]^ lithium‐sulfur batteries,^[^ ^2^ ^]^ electrocatalysis,^[^ ^3^ ^]^ and materials for absorption.^[^ ^4^ ^]^ These materials, distinguished by their unique composition and microstructure, hold promise for transformative contributions across diverse technological domains, presenting solutions to challenges with energy storage, pollution control, and catalytic processes.^[^ ^5^ ^]^ Similar to the diverse array of applications for N‐doped carbon, there are various synthetic techniques available for their preparation, such as chemical vapor deposition,^[^ ^6^ ^]^ post‐annealing of prepared carbon materials with nitrogen‐rich compounds,^[^ ^7^ ^]^ and pyrolysis of carbon precursors.^[^ ^8^ ^]^ The latter method offers significant advantages in terms of versatility, nanostructure control, purity, and scalability, making it a widespread method for synthesizing nanostructured carbon materials.^[5a]^

**Figure 1:**
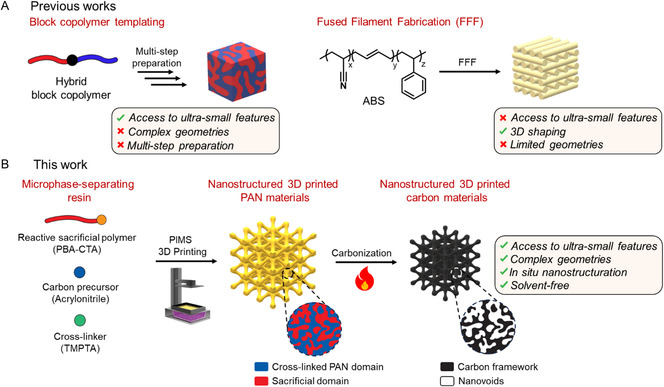
Preparation of PAN‐derived carbon materials. A) Conventional synthetic approaches (block copolymer templating and fused filament fabrication of acrylonitrile butadiene styrene (ABS) polymer) demonstrate limited capability to prepare PAN‐derived carbon materials with simultaneously controlled macro‐ and nanoscopic features. B) In this work, we report a new solvent‐free strategy to simultaneously achieve nano‐ and macroscale control of 3D printed PAN‐derived carbon materials using microphase separation 3D printing. In this process, a photocurable resin containing a reactive sacrificial polymer, a carbon precursor and a cross‐linker, i.e., poly(*n*‐butyl acrylate) with a trithiocarbonate terminus (PBA‐CTA), acrylonitrile (AN), and trimethylolpropane triacrylate (TMPTA), respectively, microphase separates and self‐assembles during 3D printing to form 3D printed materials with nanoscale morphologies composed of sacrificial polymer and cross‐linked inorganic precursor domains. Upon pyrolytic conversion, as‐printed materials are transformed into nanostructured carbon materials with isotropic shrinkage while retaining their overall macroscopic 3D architecture.

Polyacrylonitrile (PAN) is the most commonly used carbon precursor owing to its high carbon yield and unique thermal chemistry, facilitating the generation of large N‐doped graphitic domains during pyrolysis.^[8a]^ Conventionally, PAN copolymer precursors are synthesized through free radical polymerization;^[^ ^9^ ^]^ however, this method presents challenges in controlling molecular weight, dispersity, and polymer architecture. The advent of reversible–deactivation radical polymerization techniques^[^ ^10^ ^]^ has been instrumental in overcoming these limitations, enabling the production of PAN with precisely controlled molecular structures, predetermined molecular weights, low dispersity, and high chain‐end functionality.^[^ ^11^ ^]^ This has allowed the preparation of PAN with complex macromolecular architectures, such as block copolymer templated precursors,^[^ ^12^ ^]^ thus providing access to carbon‐based materials with superior properties through control over the nanoscopic morphology and pore size.^[^ ^13^ ^]^ For example, Kowalewski, Matyjaszewski et al. were the first to demonstrate the effectiveness of this strategy by utilizing ABA triblock copolymers of acrylonitrile (AN) and *n*‐butyl acrylate as a nanostructured precursor. Upon pyrolysis, the initial nanoscale morphology of the precursor was preserved, giving rise to precisely organized nanostructured carbon materials.^[13a]^ By varying the weight (wt) ratio of PAN/PBA in the precursor block copolymer, the N‐doped carbon materials displayed tunable surface area and pore size,^[12a]^ leading to their application as sorbents for CO_2_ capture.^[4a]^ However, the ultimate macro‐architectures of the carbon materials were limited to fibers and films (**Figure** **1**A). In addition, implementing time‐ and labor‐intensive protocols, such as solvent/thermal annealing, is necessary to obtain the targeted microphase‐separated morphology. This significantly increases the number of steps required for the preparation of nanostructured carbon materials.

The ability to process PAN‐based materials into intricate and complex shapes would enable the fabrication of custom‐designed carbon materials tailored to specific applications. However, PAN‐based materials are not typically prepared using digital light processing techniques, arguably one of the most utilized 3D printing methods.^[^ ^14^ ^]^ Common approaches for 3D printing of PAN‐containing materials include the use of an AN–butadiene–styrene thermoplastic polymer,^[^ ^15^ ^]^ or PAN copolymers with switchable melting temperature,^[^ ^14^ ^]^ as a feedstock for fused filament fabrication. Subsequently, the as‐printed structures are pyrolyzed to produce carbonaceous objects. However, these techniques provide carbon materials lacking nanoscale features and are constrained by the common geometric limitations inherent in extrusion‐based technologies.^[^ ^16^ ^]^ To date, current 3D printing techniques possess limited capacity to produce PAN‐derived carbon materials with independently controlled macro‐ and nanoscopic features, which can further advance this class of materials (Figure 1A).

Recent advancements in 3D printing have enabled the manipulation of material structure at both the macro‐ and nanoscale.^[^ ^17^ ^]^ Our group developed a method^[^ ^18^ ^]^ that combines the concepts of photocuring 3D printing and polymerization‐induced microphase separation (PIMS),^[^ ^19^ ^]^ a type of reaction‐induced phase transition,^[^ ^20^ ^]^ to simultaneously control both the material macro‐ and nanostructures. In this strategy, resin components, constituted by a reactive polymer, monomer, and cross‐linker, are selected in a such way that they are miscible at the onset of photopolymerization but become thermodynamically incompatible during 3D printing process, resulting in the generation of 3D printed objects with nanoscale domains. Importantly, the characteristic length scale of phase separation is restricted to the radius of gyration of the forming block copolymer. The resulting nanoscale morphology can thus be tailored by varying resin composition,^[^ 18, 21 ^]^ reactive polymer molecular weight,^[^ ^22^ ^]^ and architecture.^[^ ^23^ ^]^ By carefully selecting resin compositions and optimizing printing conditions, a diverse range of materials with distinct properties can be integrated into a single 3D printed object. This offers an accessible route to prepare functional materials, including multi‐component materials with percolating soft and hard phases with enhanced mechanical properties,^[^ ^18^ ^]^ nanostructured solid polymer electrolytes,^[19c]^ and nanoporous ceramics with morphology‐dependent thermal conduction properties.^[^ ^24^ ^]^

Herein, we present a straightforward 3D printing methodology, marking the first instance of producing PAN‐derived carbon materials with precise control over both macro‐ and nanoscales. This was achieved through the combination of liquid crystal display (LCD) printing and PIMS of photocurable resins based on a mixture of a reactive sacrificial polymer poly(*n*‐butyl acrylate) in the presence of AN and cross‐linker (Figure 1B). During the LCD 3D printing process, the photopolymerization of a resin leads to in situ microphase separation and the subsequent self‐assembly of inorganic‐precursor‐containing block copolymers, creating nanoscale morphologies in the 3D printed object in the absence of structure‐directing block copolymer templates and organic solvents. Thermal treatment of as‐printed materials enables the generation of 3D carbon structures with nanoscale voids. Importantly, the 3D printed objects maintain their macro‐ and nanoscopic architecture with isotropic shrinkage, and there is no noticeable deformation of the macrostructure upon pyrolysis. Our method offers a more accessible route to complex‐shaped carbon materials with internal nanostructuration for future applications in catalysis, energy storage, and environmental sectors.

## 2 Results and Discussion

### 2.1 Preparation and Structural Characterization of 3D Printed PAN Materials

In this study, poly(*n*‐butyl acrylate) with a trithiocarbonate terminus (PBA‐CTA) was selected as a macromolecular chain transfer agent (macroCTA) due to its solubility in AN and trimethylolpropane triacrylate (TMPTA). PBA‐CTA was synthesized by reversible addition‐fragmentation chain transfer (RAFT) polymerization using 2‐(*n*‐butylthiocarbonothioylthio) propanoic acid as RAFT agent and 2,2´‐azobisisobutyronitrile as thermal initiator (Figure S1 and Table S1, Supporting Information). The degree of polymerization (*X*_*n*_) and the number‐average molecular weight (*M*_*n*_) for the purified macroCTA were 186 and 24 100 g mol^−1^, respectively, as determined by proton nuclear magnetic resonance spectroscopy (Figure S3, Supporting Information), while the dispersity (*Đ*) was 1.09 as determined by size‐exclusion chromatography (Figure S4, Supporting Information). AN was selected as the monomer to generate PAN, which is thermodynamically incompatible with PBA_186_‐CTA,^[12a]^ and produces carbon in high yield upon pyrolysis.^[8a]^ TMPTA, commonly used in UV‐curable coatings, was chosen as a cross‐linker for the preparation of the polymeric network. Diphenyl(2,4,6‐trimethylbenzoyl) phosphine oxide, a Norrish Type I photoinitiator, was utilized for its ability to effectively initiate RAFT polymerization during open‐to‐air 3D printing.^[^ ^18^ ^]^ In addition, a small amount of a light‐absorbing dye Sudan II (0.03 wt%) was added to the resins to avoid gelation outside of the printing area to achieve high‐quality prints (Figure S5, Supporting Information). Subsequently, homogeneous, transparent resins were formulated by mixing AN, TMPTA, PBA_186_‐CTA, and TPO in predetermined wt ratios (Figure S6, Supporting Information). The concentrations of PBA_186_‐CTA and TPO were kept constant, specifically at 28.8 and 1.0 wt%, while the molar ratio of [AN]/[TMPTA] varied between 20/1, 40/1, 100/1, and 500/1 (Table S2, Supporting Information). The formulated resins were used to 3D print rectangular prisms using a commercial LCD 3D printer (Anycubic Photon Mono SE, *λ*_max_ = 405 nm, *I*_0_ = 2.0 mW cm^−2^) with a layer slicing thickness of 50 μm and a cure time of 110 s per layer. It should be noted that the variations in the molar ratio of [AN]/[TMPTA] did not affect the macroscopic resolution of 3D printed objects.

**Figure 2:**
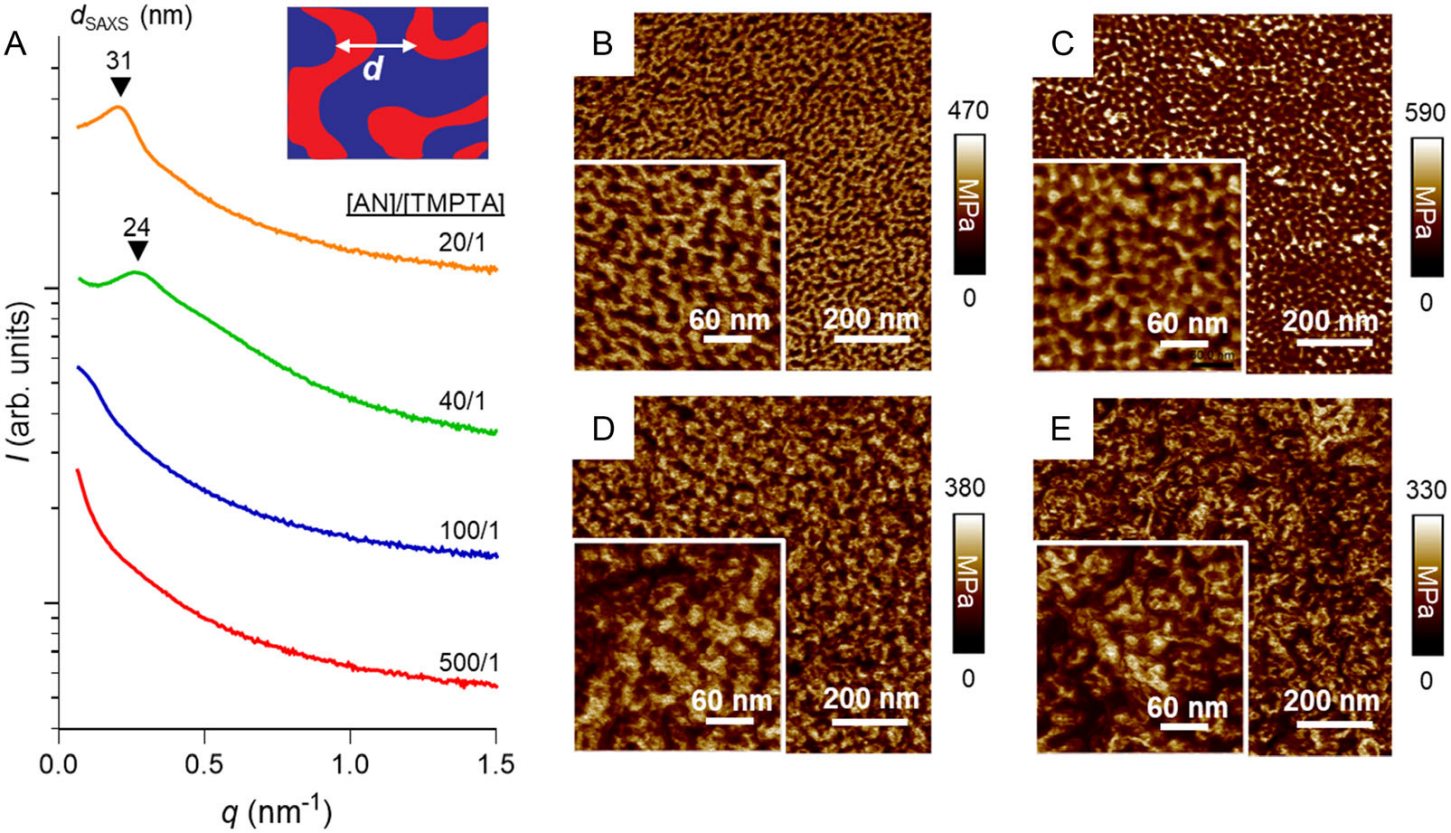
Characterization of 3D printed PIMS PAN materials. A) SAXS profiles and corresponding domain spacing (*d*_SAXS_) values of PAN 3D printed materials with [AN]/[TMPTA] = 20/1, 40/1, 100/1, and 500/1 and 28.8 wt% of PBA_186_‐CTA loading. SAXS profiles were shifted vertically for clarity. B–E) AFM images of nanoscale morphology for PAN materials 3D printed with [AN]/[TMPTA] = (B) 20/1, (C) 40/1, (D) 100/1, and (E) 500/1. PBA and *net*‐P(AN‐*stat*‐TMPTA) domains are shown in dark brown and light brown, respectively.

During the 3D printing process, PBA‐CTA is chain extended with AN and TMPTA to form cross‐linked network copolymers with PBA and *net*‐P(AN‐*stat*‐TMPTA) segments. These block copolymers have a large Flory–Huggins interaction parameter (*χ* ≈ 1.711 at 25 °C, Supporting Information), which indicates their pronounced tendency to microphase separate. During the printing process, the incompatible blocks within the copolymer undergo microphase separation to minimize energetically unfavorable interactions between distinct segments. The emergent nanoscale morphology is ultimately arrested by in situ cross‐linking. The 3D printed PAN materials were characterized by small‐angle X‐ray scattering (SAXS) to investigate their internal nanoscale structures (Figure S7, Supporting Information). The SAXS spectra of the PAN materials 3D printed with [AN]/[TMPTA] = 20/1 and 40/1 and PBA_186_‐CTA loading of 28.8 wt% exhibited a single broad peak at 0.203 and 0.262 nm^−1^, corresponding to center‐to‐center domain spacings (*d*_SAXS_) of 31 and 24 nm, respectively (**Figure** **2**A). The broad SAXS peak indicates the generation of a disordered microphase‐separated morphology that lacks long‐range order.^[^ 18, 19, 22, 25 ^]^ The fitting of SAXS spectra using the Teubner–Strey (T–S) model^[^ ^26^ ^]^ extracted two parameters, i.e., the amphiphilicity factor (*f*_a_) and the domain size polydispersity (*ξ*/*d*_TS_) (Table S3, Supporting Information). The *f*_a_ and *ξ*/*d*_TS_ values for PAN materials 3D printed with [AN]/[TMPTA] = 20/1 and 40/1 were −0.81 and −0.74 and 0.49 and 0.42, respectively, suggesting the generation of well‐structured domains with sharp interfaces and broad dispersity. In contrast, in the case of PAN materials 3D printed with [AN]/[TMPTA] = 100/1 and 500/1, the SAXS spectra showed no discernible peak in the measured range, suggesting that the domain spacing for these samples is beyond the detection limit of the SAXS instrument (Figure 2A).

The nanostructure of 3D printed PAN materials was further confirmed by atomic force microscopy (AFM) using the PeakForce quantitative nanomechanics mode. Materials 3D printed with [AN]/[TMPTA] = 20/1 and 40/1 displayed co‐continuous nanoscale hard PAN and soft PBA networks (Figure 2B,C). Analysis of AFM images revealed that the average PBA domain width and domain spacing changed from 13 to 14 nm and from 31 to 25 nm, respectively, upon varying [AN]/[TMPTA] from 20/1 to 40/1. Importantly, the domain spacing determined by AFM is in close agreement with the average interdomain distance determined by SAXS, thereby supporting the reliability of the AFM imaging. Further increasing the molar ratio of [AN]/[TMPTA] to 100/1 and 500/1 resulted in the generation of ill‐defined nanoscale morphologies (Figure 2D,E).

### 2.2 Stabilization and Carbonization of 3D Printed PAN Materials

**Figure 3:**
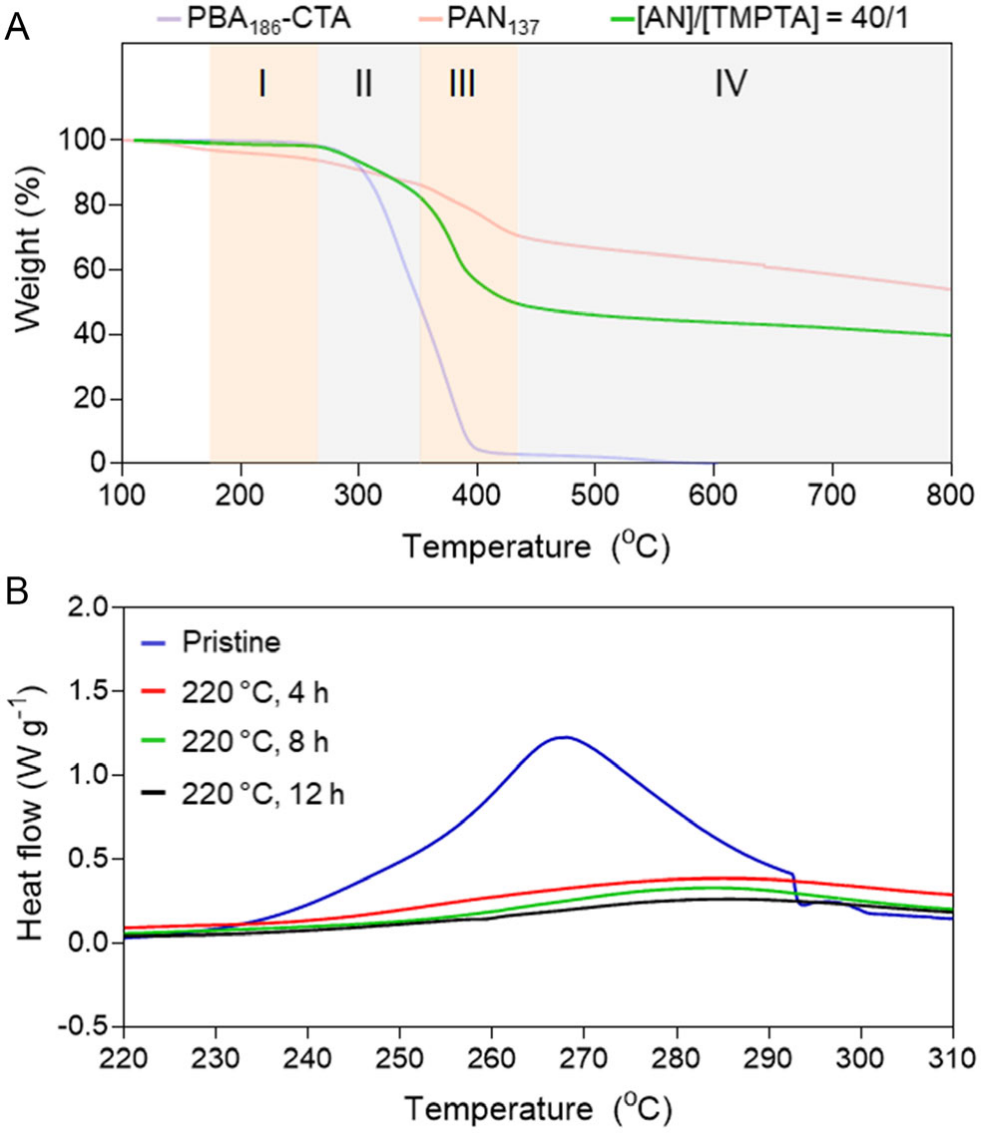
Thermal characterization of 3D printed PIMS PAN materials. A) TGA profiles of PBA_186_‐CTA, PAN_137_, and the 3D printed PAN materials prepared with the molar ratio of [AN]/[TMPTA] = 40/1 and 28.8 wt% of PBA_186_‐CTA. The heating rate was 10 °C min^−1^ under nitrogen. The 4 distinct stages were observed for 3D printed PAN materials prepared using [AN]/[TMPTA] = 40/1: stages I (170–270 °C), II (270–350 °C), III (350–420 °C), and IV (>420 °C). Please see the main text for the description of stages; B) DSC thermograms of pristine and stabilized PAN materials 3D printed with the molar ratio [AN]/[TMPTA] = 40/1 and 28.8 wt% of PBA_186_‐CTA.

The processing of PAN into carbon is a two‐step process that includes oxidative stabilization of the PAN phase at moderate temperatures (200–300 °C) to form a ladder polymer, followed by carbonization in an inert atmosphere at 600–1300 °C.^[8a]^ To find temperature conditions for the stabilization stage, PBA_186_‐CTA, PAN_137_ (Table S1 and Figure S1–S4, Supporting Information), and 3D printed PAN materials were characterized by thermogravimetric analysis (TGA) (**Figure** **3**A). The TGA profile of PBA_186_‐CTA in a nitrogen atmosphere showed a significant weight loss of 97 wt% in the temperature range of 240–410 °C and full degradation at 560 °C. In contrast, PAN_137_ displayed a weight loss of 30 wt% at 170–440 °C due to dehydrogenation reactions and cyclization of the nitrile groups.^[^ ^27^ ^]^ Gradual weight loss beyond 440 °C was attributed to further cyclization and dehydrogenation resulting in a carbon yield of 54 wt%, which aligns with the previous works on PAN.^[12b]^ The TGA profiles of 3D printed PAN materials revealed four distinct weight loss stages (Figure 3A and S8, Supporting Information). The first stage occurred in the temperature range of 170–270 °C and can be attributed to the dehydrogenation and cross‐linking of PAN. The second stage from 270 to ≈350 °C reflected further cross‐linking of PAN phase and the onset of thermal degradation of PBA phase. Further heating to ≈420 °C resulted in rapid weight loss due to the complete decomposition of PBA. The TGA curves exhibited gradual weight loss at temperatures above 420 °C associated with further cyclization and dehydrogenation of PAN phase, yielding ≈40 wt% of carbon, which is close to the theoretical values for PAN samples containing 28.8 wt% of PBA. Based on the TGA results, we selected a temperature of 220 °C for the stabilization stage in order to achieve cross‐linking of PAN phase to stabilize the microphase separated morphology before the onset of PBA thermal degradation.

**Figure 4:**
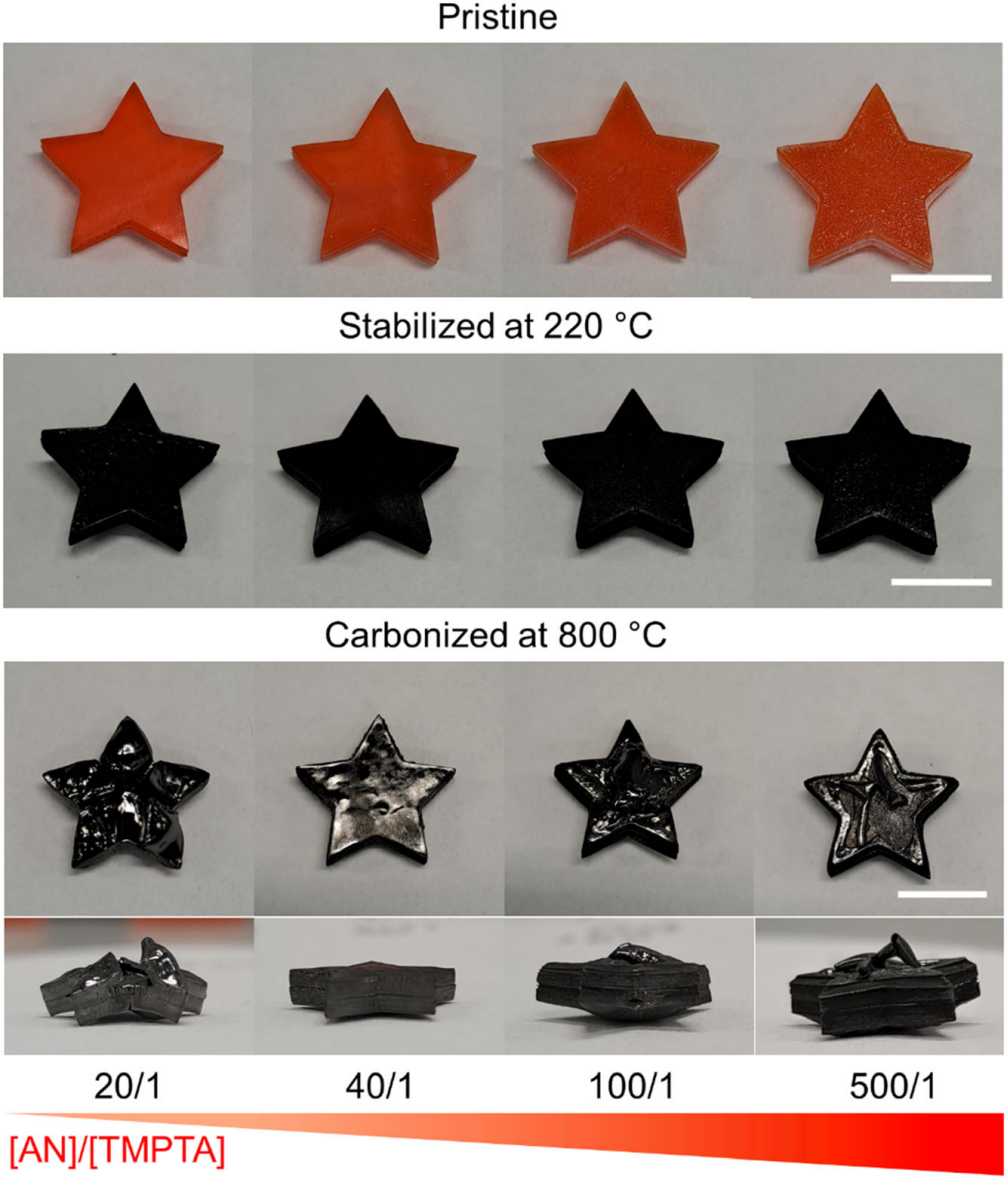
Photographs of pristine, stabilized (at 220 °C) and carbonized (at 800 °C) PIMS materials 3D printed with various molar ratio of [AN]/[TMPTA]. Scale bars for the pristine and stabilized samples are 10 mm. Scale bar for carbonized samples is 8 mm.

The stabilization of 3D printed PAN materials was carried out by heating from ambient temperature to 220 °C in air at a rate of 1 °C min^−1^. The color of samples changed from orange to black, indicating both the dehydrogenation of PAN and cyclization of the nitrile groups to form a ladder‐like, conjugated polymer,^[^ ^14^ ^]^ as well as partial degradation of Sudan II dye (**Figure** **4**).

During the stabilization stage, the weight loss and shrinkage of the samples were 2–3 and 5–7%, respectively. To evaluate the efficiency of the oxidative stabilization of the PAN phase, we performed differential scanning calorimetry (DSC) of the 3D printed materials stabilized at 220 °C for 4, 8, and 12 h. The DSC thermogram of PAN_137_ recorded from 200 to 310 °C exhibited an exothermic peak attributed to the cross‐linking of nitrile groups via cyclization, while PBA_186_‐CTA displayed the onset of an endotherm assigned to its gradual decomposition (Figure S9A, Supporting Information). The pristine 3D printed materials showed the characteristic PAN exotherm; however, the peak position shifted to higher temperature and the sharpness of the exothermic peak decreased as the cross‐linking density of materials increased (Figure 3B and S9B–D, Supporting Information). The materials treated at 220 °C under air for 4 h demonstrated significant reduction in the intensity of the exothermic peak. As the treatment time increased to 8 h, the exothermic peak was no longer observed for the materials 3D printed with [AN]/[TMPTA] = 100/1 and 500/1, while a further decrease in the peak intensity was observed for the materials prepared with [AN]/[TMPTA] = 20/1 and 40/1. Extending the treatment time to 12 h did not result in significant changes in DSC thermograms of the materials. Altogether, these results indicate that the cross‐linking process within the majority of the *net*‐P(AN‐*stat*‐TMPTA) phase has already taken place.

To perform the carbonization process, the materials were pyrolyzed in a nitrogen atmosphere at 800 °C with a heating rate of 1 °C min^−1^. Among all samples, only materials 3D printed with a molar ratio of [AN]/[TMPTA] = 40/1 maintained its macroscopic shape without any observable deformation (Figure 4). This may be attributed to the insufficient cross‐linking of the PAN phase during the stabilization stage for the material prepared with [AN]/[TMPTA] = 20/1 and the low degree of structural support provided by the lower amount of cross‐linker, e.g., [AN]/[TMPTA] = 100/1 and 500/1. For the material prepared with [AN]/[TMPTA] = 40/1, the carbonization resulted in a shrinkage of the printed object by 25% and a carbon yield of 42 wt%, which is closely aligned with the TGA data (Figure 3A). Furthermore, the macroscopic architecture of 3D printed objects was maintained upon carbonization at 1000 and 1200 °C (Figure S10, Supporting Information).

### 2.3 Chemical and Structural Transformations during Thermal Treatment of 3D Printed PIMS PAN Materials

**Figure 5:**
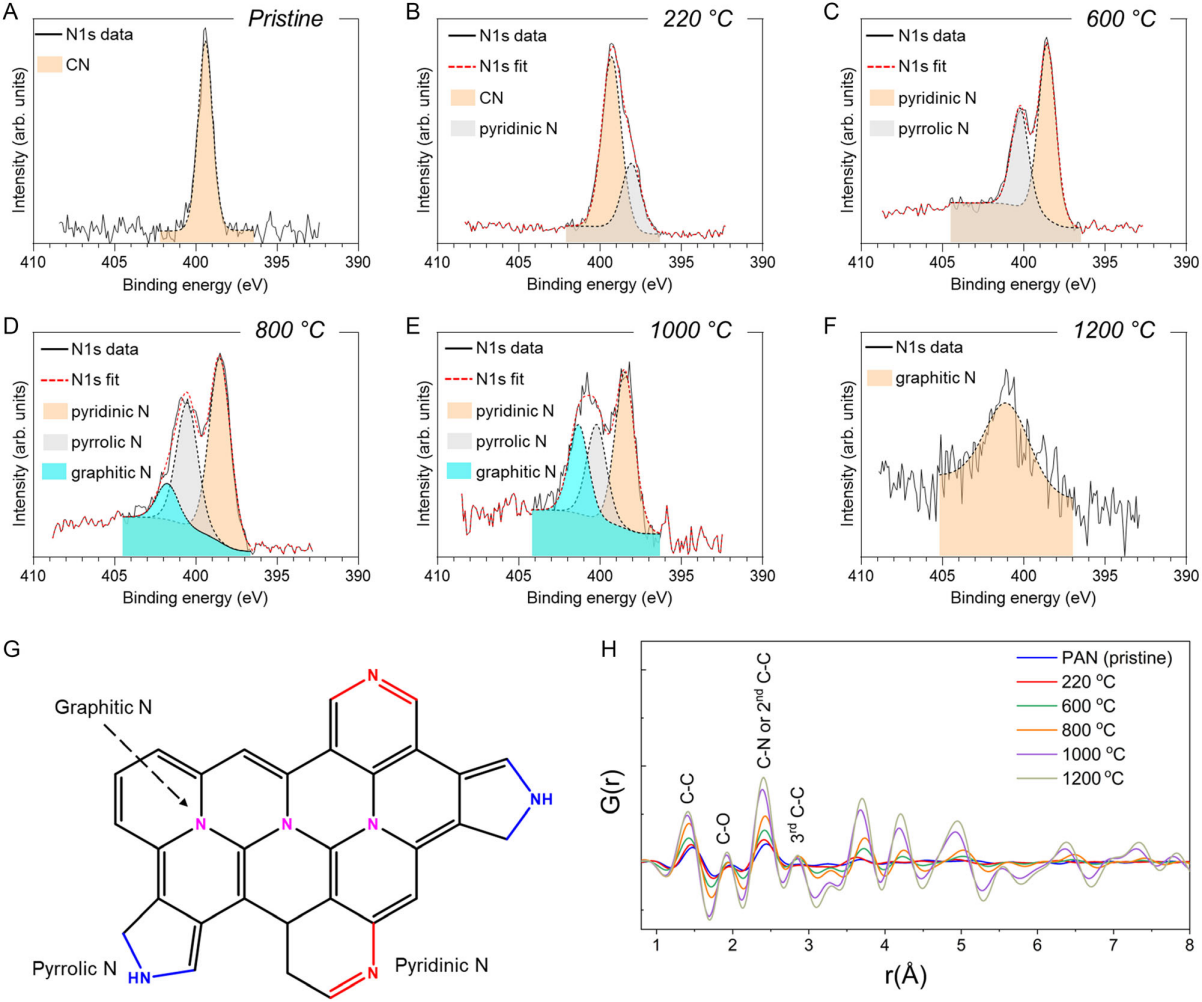
Compositional and structural analysis of 3D printed PIMS PAN materials at different stages of pyrolysis. A–F) Deconvoluted N1s XPS spectra of 3D printed PIMS PAN materials that are (A) pristine, or treated at (B) 220, (C) 600, (D) 800, (E) 1000, and (F) 1200 °C. G) A chemical structure of possible N species formed during PAN pyrolysis. H) Atomic PDFs of 3D printed PIMS PAN material at different stages of ex situ pyrolysis, shown up to 8 Å. The material was 3D printed using the molar ratio of [AN]/[TMPTA] = 40/1 and 28.8 wt% PBA_186_‐CTA.

The pyrolysis temperature at which the PAN thermal transformation is carried out can significantly affect the resulting material properties.^[^ ^28^ ^]^ To investigate this, electronic conductivity of the materials as a function of treatment temperature was measured. A 4‐point probe technique revealed a clear trend of increasing electronic conductivity with higher carbonization temperatures (Figure S11, Supporting Information). Specifically, the material transitioned from nonconductive to highly conductive (2.5 × 10^−2^ S cm^−1^) after pyrolysis at 1200 ºC, which is comparable the conductivity reported for other PAN‐derived carbon materials.^[^ ^28^ ^]^ This conductivity increase can be attributed to the increased degree of graphitization and the variations in chemical compositions upon thermal transformation of PAN to carbon. During pyrolysis, the materials undergo significant graphitization, as indicated by Raman spectra at different stages of the process (Figure S12, Supporting Information). These spectra closely resembled those found in existing literature on PAN‐derived carbon materials,^13^, ^28^ with a characteristic strong, relatively narrow band at 1575 cm^−1^, representing a graphitic form (G‐band, *sp* ^2^ carbon), and a broader peak at 1355 cm^−1^, corresponding to *sp* ^3^ carbon species (D‐band). The D‐ to G‐band intensity ratio, which indicates the degree of graphitization, decreased from 0.98 to 0.94 as the carbonization temperature increased from 600 to 1200 °C (Figure S12, Supporting information). This indicates a higher contribution of graphitic species in the structural arrangement of carbon materials, resulting in increased electrical conductivity.^[^ ^28^ ^]^ X‐ray photoelectron spectroscopy (XPS) was also conducted to monitor the chemical changes at different stages of pyrolysis of 3D printed PIMS PAN materials (**Figure** **5**A–G). The N1*s* XPS spectra clearly indicate variations in nitrogen composition. It is known that upon thermal transformation from PAN to N‐doped carbon, nitrogen can exist as pyridinic, pyrrolic, and graphitic species (Figure 5G).^[8a]^ In the 3D printed pristine sample, N exists exclusively in the form of the CN nitrile species (Figure 5A). After the stabilization stage at 220 °C, 71% of the N species still exist in the CN form, with pyridinic N accounting for the remaining 29% of the total N species (Figure 5B). Pyridinic N typically originates from the cyclization of the CN groups.^[8a]^ At 600 °C, the CN groups are fully converted to edge‐site N functionalities, comprising 63% pyridinic N species and 36% pyrrolic N species (Figure 5C). The carbonization at higher temperatures, e.g., 800 and 1000 °C, resulted in the generation of three N species, that is, pyridinic N, pyrrolic N, and graphitic N (Figure 5D,E). The content of graphitic N increased from 10 to 26%, while the contents of pyridinic and pyrrolic species decreased from 53 and 36 to 46% and 28%, respectively, as the carbonization temperature was raised from 800 to 1000 °C. A further increase in the carbonization temperature to 1200 °C led to complete decomposition of pyridinic and pyrrolic groups due to their high temperature instability,^[^ ^29^ ^]^ resulting in the formation of only graphitic N (Figure 5F). It should be noted that the N/C ratio decreased significantly from 0.09 to 0.015 as the pyrolysis temperature increased from 800 to 1200 °C due to denitrogenation occurring at high temperatures.^[^ ^30^ ^]^ For carbon compositions, the comparison of C1*s* XPS spectra obtained for pristine and thermally treated materials revealed that C largely exists in the form of C‐*sp* ^3^ and C‐*sp* ^2^, as well as pyridinic and pyrrolic configurations (Figure S13, Supporting information). However, minor contributions from C─O, C═O, and O–C═O oxidized species were observed, and their presence decreased as the carbonization temperature increased (Figure S13, Supporting Information). This was attributed to the presence of residual PBA in the materials, which further degraded during the pyrolysis process.

As XPS only provides information on the chemical composition on the surface of materials, the internal structural changes of 3D printed PAN materials was further investigated using high‐energy X‐ray diffraction (HE‐XRD) coupled with pair distribution function (PDF) analysis, which is a powerful method to determine atomic arrangements in nanostructured materials exhibiting any degree of structural coherence and periodicity.^[^ ^31^ ^]^ The PDF analysis allowed us to identify distinct atomic contributions, specifically C—C (1.42–1.46 Å), C—O (1.93–1.95 Å), C—N (2.43–2.45 Å), and the 2^nd^ graphite C—C (≈2.40 Å) and 3^rd^ graphite C—C (≈2.86 Å) interactions (Figure 5H).^[^ ^32^ ^]^ Initially, the pristine material displayed a predominant amorphous structure characterized by two primary peaks corresponding to the nearest neighbor C—C and C—N distances, typically associated with the intra‐chain arrangement in the PAN structure.^[32b]^ Another discrete peak at approximately 1.93 Å consistently appeared for materials treated at all temperatures, corresponding to either backscattering signals from C—C and/or C—O contributions from oxidized PAN. Upon heating to 220 °C, the material maintained a similar amorphous structure to the pristine sample, with minor changes observed in the C—N interatomic distances, which shifted to a shorter *r* value by approximately 0.05 Å, likely due to the initial cyclization process. Another discrete peak at approximately 1.93 Å consistently appeared for the materials pyrolyzed from 220 to 800 °C, corresponding to C—O contributions from oxidized PAN. Notably, this peak gradually shifts to lower *r* distances, more closely assembling the backscattering contributions of C—C in graphitic structures. It is worth noting that during dehydrogenation, oxidation, and cyclization reactions, functional groups such as C═C, C═O, and C═N were formed.^[^ 32, 33 ^]^ Significant transformations in the local structure became apparent as the temperature reached 600 °C, predominantly impacting the first coordination shell of C—C atomic pairs, resulting in a prominent shift from 1.46 to 1.43 Å. Moreover, pyrolysis at 600 °C led to the emergence of a new peak at 2.86 Å, intensifying with further heating. This peak corresponds to the third neighbor distance in graphitic structures (≈2.84 Å) and suggest the formation of planar six‐member ring structures, which is in accord the results obtained by Raman spectroscopy.^[32b]^ It is important to note that during carbonization, the linear chains underwent a complete conversion into cyclized ladder‐like structures, leading to the third neighbor distance closely mirroring that of an ideal graphite crystal.^[32b]^ Upon reaching 800 °C, while sharper main peaks indicated enhanced structural order, the local structural characteristics remained similar to those observed at 600 °C. Further heating to 1000 °C resulted in the first C—C contribution shifting to 1.42 Å, closely resembling the ideal crystal structure of graphite in the first coordination shell. We also observed additional shifts in the second primary peak, ranging from 2.45 Å at 220 °C to 2.42 Å at 800 °C. This shift was attributed to the interatomic distances involving C—N pairs. At 1000 °C and beyond, this peak appeared at 2.40 Å, which is close to the second neighbor distance of C—C in the graphite structure.^[^ 32, 33 ^]^ Prior investigations into N‐doped carbon networks have revealed great similarities in bond lengths for the first and second peaks, as compared to pure carbon structures.^[^ ^34^ ^]^ Consequently, based on XPS findings, it is plausible that the second‐nearest neighbor distance may also be originated from C—N contributions within N‐containing structures, particularly at 800 and 1000 °C. Overall, the atomic PDFs revealed a progressive increase in the degree of order as the carbonization temperature was raised, resulting in the shortening of the primary interatomic distances, a phenomenon consistent with previous findings in PAN carbonization.^[32b]^ To provide a more comprehensive view of the long‐range structural changes, we have included atomic PDFs spanning a range of up to 20 Å, highlighting well‐defined peaks associated with PAN graphitization, notably pronounced at 1000 °C and beyond (Figure S14, Supporting Information).

### 2.4 Fabrication of Complex‐Shaped Carbon Architecture

**Figure 6:**
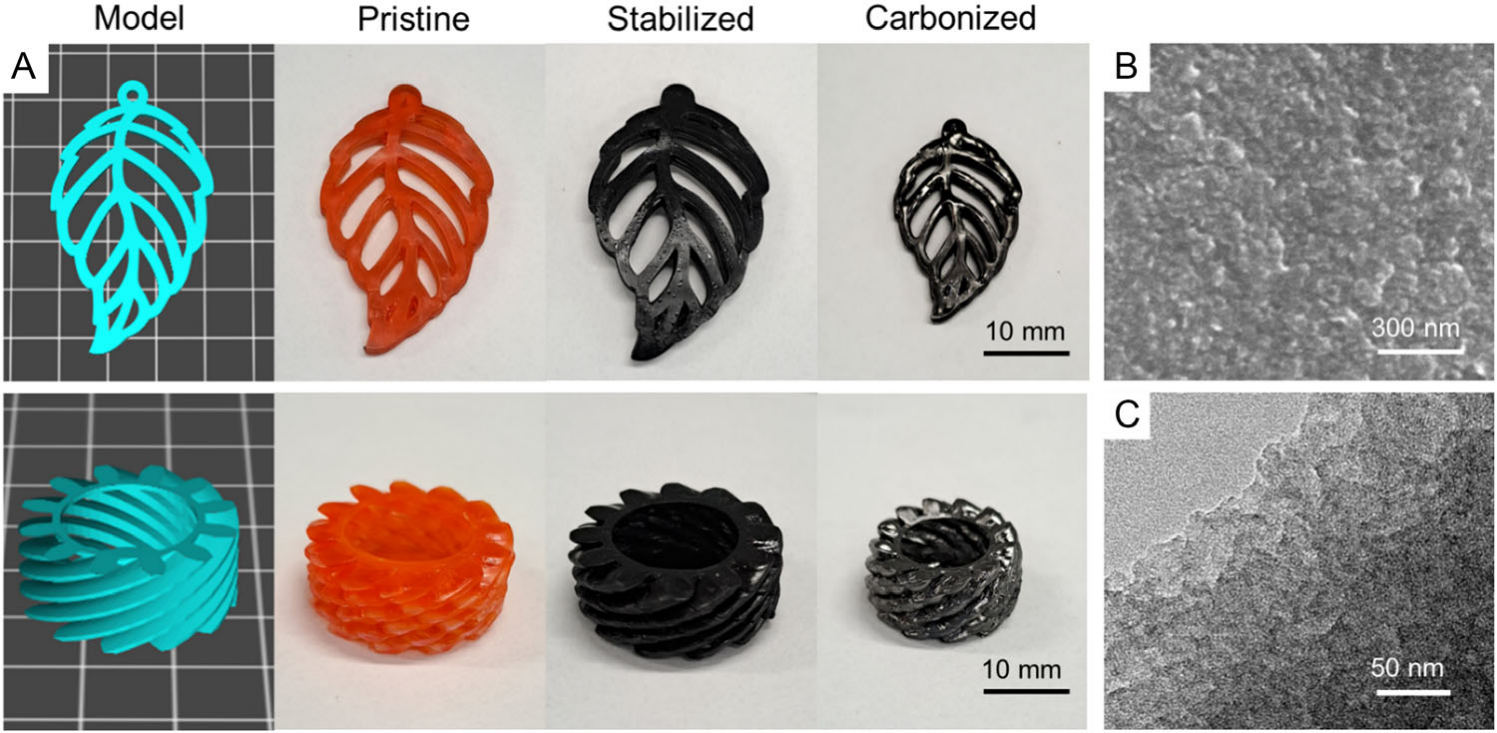
Complex‐shaped objects and microstructural characterization of 3D printed PIMS PAN materials. A) Computer‐aided design models and photographs of pristine, stabilized, and carbonized 3D printed complex objects (a leaf and a gear part). The materials were 3D printed using the molar ratio of [AN]/[TMPTA] = 40/1 and 28.8 wt% PBA_186_‐CTA. Protocol for stabilization stage: heating from ambient temperature to 220 °C at a heating rate of 1 °C min^−1^ in air and held at 220 °C for 1 h. Protocol for carbonization: heating from ambient temperature to 800 °C at a heating rate of 1 °C min^−1^ in nitrogen and held at 800 °C for 1 h; B) SEM and C) TEM micrographs (cross‐section) of a carbonized object (800 °C, under nitrogen).

To showcase the effectiveness of our approach, we shifted our focus toward creating carbon materials with complex macroscale architectures that are challenging to produce via traditional manufacturing approaches. By using this optimized resin formulation and pyrolysis conditions, we were able to successfully 3D print complex‐shaped PAN structures (leaf‐ and gear‐shaped objects) and convert them into carbon materials while preserving their overall macroscopic shapes (**Figure** **6**A).

The leaf‐ and gear‐shaped objects were designed to have dimensions of 25 × 40 × 3.5 mm and 25 × 25 × 10 mm, respectively. The 3D printed PAN materials replicated the original CAD models with high printing fidelity. Specifically, the measured dimensions for leaf‐ and gear‐shaped objects were 25.3 × 40.4 ×3.7 mm and 25.4 × 25.6 × 10.0 mm, respectively, which is in close agreement with the expected values. Remarkably, the 3D printed objects provided a faithful reproduction of a midrib and veins and helical teeth for leaf‐ and gear‐shaped objects, respectively. After the stabilization stage, the leaf‐ and gear‐shaped materials lost 3% and 7% of their mass, while their sizes were reduced by 6% and 8%, respectively. The subsequent carbonization step induced a significant contraction of the printed objects (≈24%), while yielding a carbon content of ≈45 wt%. Examination of the cross‐sectional area of the resulting materials by scanning and transmission electron microscopy revealed the presence of an interconnected carbon matrix with embedded nanoscale voids after pyrolytic removal of the sacrificial PBA block (Figure 6B,C). The SAXS analysis of the carbonized material revealed a broad peak with a *d*_SAXS_ ≈ 5 nm, which could correspond to the mesopore size (Figure S15, Supporting Information). Energy‐dispersive X‐ray spectroscopy revealed a uniform distribution of C, O, and N elements throughout the bulk material (Figure S16, Supporting Information). In addition, XPS analysis confirmed the presence of N‐containing species, namely pyridinic, pyrrolic, and N‐graphitic (Table S4, Supporting Information). These functional groups can be utilized to enhance the adsorption of gas and water,^[^ ^35^ ^]^ as well as improve the performance of carbon materials in supercapacitors.^[^ ^36^ ^]^ These results demonstrate the design flexibility of our approach to fabricate nanostructured N‐doped carbon materials of various 3D shapes.

## 3 Conclusion

In summary, we have developed a novel approach for LCD 3D printing of nanostructured carbon materials derived from PAN using a commercially available 3D printer. This was enabled by utilizing a photocurable resin capable of undergoing microphase separation and self‐assembly upon polymerization, resulting in the generation of 3D printed materials with nanoscale morphologies. These materials consist of distinct domains of carbon precursors, i.e., PAN, and a sacrificial polymer. Through understanding of the intricate interplay among resin composition, printing conditions, and pyrolysis process parameters, carbon materials with controlled structures spanning from the nano‐ to macroscale were prepared in a straightforward manner. The findings of this work will facilitate the development of a rich variety of carbon‐containing structures with customized geometries for a range of diverse prospective applications including catalysis, energy storage, electronics, and conductive materials.

## Conflict of Interest

The authors declare no conflict of interest.

## Supporting information

Supplementary Material

## Data Availability

The data that support the findings of this study are available from the corresponding author upon reasonable request.
