# Supplementary material for: Design and 3D Printing of Polyacrylonitrile‐Derived Nanostructured Carbon Architectures
Source: Small Sci. 2024 Feb 27;4(4):2300275. doi: 10.1002/smsc.202300275 (PMC11935245; doi:10.1002/smsc.202300275)
Supplement: Supplementary file 1 — Supplementary Material [file SMSC-4-2300275-s001.pdf]

Supporting Information

Design and 3D Printing of Polyacrylonitrile-Derived Nanostructured Carbon Architectures

Valentin A. Bobrin,[*, 1] Haira G. Hackbarth,[2] Yin Yao,[3] Dipan Kundu,[2] Nicholas M. Bedford,[2] Rhiannon P. Kuchel,[3] Jin Zhang[*, 4], Nathaniel Corrigan[*, 1], Cyrille Boyer[*, 1, 5]

[1]Cluster for Advanced Macromolecular Design, School of Chemical Engineering, University of New South Wales, Sydney, NSW 2052, Australia

[2]School of Chemical Engineering, University of New South Wales, Sydney, NSW 2052, Australia

[3]Electron Microscope Unit, Mark Wainwright Analytical Centre, University of New South Wales, Sydney, NSW 2052, Australia

[4]School of Mechanical and Manufacturing Engineering, University of New South Wales, Sydney, NSW 2052, Australia

[5]Australian Centre for Nanomedicine, School of Chemical Engineering, University of New South Wales, Sydney, NSW 2052, Australia

*Email: cboyer@unsw.edu.au; n.corrigan@unsw.edu.au; jin.zhang6@unsw.edu.au; v.bobrin@unsw.edu.au.

Materials and Methods

Materials

Unless otherwise stated, all chemicals were used as received. The solvents were of either HPLC or AR grade; these included acetonitrile (RCI Labscan Limited, RCI Premium) and tetrahydrofuran (THF, RCI Labscan Limited, HPLC). Aluminium oxide basic (Acros Organics, Brockmann I, 50–200 µm, 60A), 2-(*n*-butylthiocarbonothioylthio)propanoic acid (BTPA, Boron Molecular, >95%), 4-cyano-4-(((dodecylthio)carbonothioyl)thio)pentanoic acid) (CDTPA, Boron Molecular, 99%), diphenyl (2,4,6-trimethylbenzoyl) phosphine oxide (TPO, Sigma-Aldrich, ≥70%), 2,2'-azobis(2-methylpropionitrile) solution (AIBN solution, Sigma-Aldrich, 0.2M in toluene), acrylonitrile (AN, Sigma-Aldrich, ≥99%) and trimethylolpropane triacrylate (TMPTA, Sigma-Aldrich, technical grade, 80%) were used as received. *n*-Butyl acrylate (BA, Sigma-Aldrich, technical grade, ≥99%) was passed through a basic aluminium oxide column to remove inhibitor prior to use.

Characterization methods

Nuclear magnetic resonance (NMR)

All NMR spectra were recorded on Bruker Avance III 400 MHz spectrometer using an external lock (toluene-d_8 or DMSO-d_6).

Size exclusion chromatography (SEC)

Analysis of the molecular weight distributions of the polymers were determined using a Shimadzu modular system composed of an SIL-20A auto-injector, a Polymer Laboratories 5.0 µm bead-size guard column (50 × 7.5 mm^2) followed by three linear PL (Styragel) columns (10^5, 10^4 and 10^3 Å), an RID-10A differential refractive-index (RI) detector, and a UV detector. The eluent was DMAc (containing 0.03% w/v LiBr and 0.05% w/v 2,6-dibutyl-4-methylphenol (BHT)) at 50°C, run at a flow rate of 1.0 mL/min. The SEC was calibrated using narrow polystyrene (PSTY) standards with molecular weights of 200 – 10^6 g/mol.

Estimation of $\chi_{\text{P(AN-stat-TMPTA)-b-PBA}}$ by group molar contribution method

$\chi_{\text{P(AN-stat-TMPTA)-b-PBA}}$ was estimated using equation S1:

$$\chi_{\text{P(AN}-stat-\text{TMPTA)}-b-\text{PBA}} = (1-x)\chi_{\text{TMPTA}-\text{PBA}} + x\chi_{\text{PAN}-\text{PBA}} + x(1-x)\chi_{\text{PAN}-\text{TMPTA}} \quad \textbf{(Eq. S1)}$$

where x is the weight fraction of AN in P(AN-*stat*-TMPTA) block (x = 0.88 for the molar ratio [AN]/[TMPTA] = 40/1). χ_{12} was calculated using equation S2:

$$\chi_{12} = \frac{VN_A}{RT}(\delta_1 - \delta_2)^2 \quad \textbf{(Eq. S2)}$$

where V is the reference volume (set to 118 Å^3), R is the gas constant (1.987 cal mol^{-1} K^{-1}), T is temperature (set to 298 K), N_A is the Avogadro's number (6.02 × 10^{23} mol^{-1}), δ ((cal cm^{-3})$^{1/2}$) is solubility parameter estimated using the group molar contribution method proposed by Small[1] (equation S3):

$$\delta = \frac{d\Sigma G}{M} \quad \textbf{(Eq. S3)}$$

where d (g cm^{-3}) is density, M is monomer molecular weight, $\sum G$ is the sum of the molar attraction constants. Estimated δ values were as follows: δ_{PBA} = 9.15 $cal^{1/2}$ $cm^{-3/2}$, δ_{PAN} = 12.74 $cal^{1/2}$ $cm^{-3/2}$, δ_{TMPTA} = 7.74 $cal^{1/2}$ $cm^{-3/2}$. Then, χ parameters were calculated using equation S2: $\chi_{TMPTA\text{-}PBA}$ = 0.238, $\chi_{PAN\text{-}PBA}$ = 1.551, $\chi_{PAN\text{-}TMPTA}$ = 3.004. Subsequently, $\chi_{P(AN\text{-}stat\text{-}TMPTA)\text{-}b\text{-}PBA}$ was calculated using equation S1: $\chi_{P(AN\text{-}stat\text{-}TMPTA)\text{-}b\text{-}PBA}$ = 1.711.

Atomic force microscopy (AFM)

All AFM measurements were performed on the Bruker Dimension ICON SPM, with a Nanoscope V controller (software version 9.70). Mechanical property measurements were performed using peak force tapping mode on the top layer of printed object using the SCANASYST probe (from www.brukerafmprobes.com). The scan size was set to 1 µm and 300 nm. The scan rate was set at around 0.7 to 0.8 Hz with a peakforce of approximately 500 pN. The feedback gain was adjusted accordingly to optimize tracking of the specimen surface. The resolution of the image was set to 512 pixels per line for 1 µm scan size and 256 samples/line for 300 nm scan size. For peakforce mechanical measurements, the tip was calibrated using the thermal tunning method. AFM images were analysed using NanoScope Analysis software, version 1.7.

Small-angle X-ray scattering (SAXS)

SAXS experiments were performed on an Anton Paar SAXSPoint 2.0 system with a Cu K_α (λ = 0.154 nm) microfocus X-ray source (50 kV/1 mA) and Dectris Eiger 1M detector. Data was collected at room temperature, under vacuum for 5 min from a sample at a sample-to-detector distance of 0.575 m. Samples were 3D printed at the thickness of 2×100 µm layers. Data was reduced to 1D by radial averaging the 2D detector after converting pixel positions to $q = (4\pi/\lambda)\sin\theta$, where 2θ is the scattering angle). The domain spacing was calculated using equation S4:

$$d_{SAXS} = \frac{2\pi}{q} \quad \textbf{(Eq. S4)}$$

SAXS fitting using Teubner-Strey (T-S) model

The position and the sharpness of SAXS peaks of microphase-separated 3D printed preceramic materials were fitted using T-S model in SasView software. According to T-S model (equation S5)

$$I(q) = \frac{1}{a_2 + c_1 q^2 + c_2 q^4} + b \quad \textbf{(Eq. S5)}$$

Where $q = (4\pi/\lambda)sin\theta$, λ is the wavelength, 2θ is the scattering angle; b is background scattering; a_2, c_1, c_2 are fitting parameters used to calculate domain spacing (d_{TS}), correlation length (ξ) and the amphiphilicity factor (f_a) using equations S6-8 below:

$$d_{TS} = 2\pi\left[\frac{1}{2}\left(\frac{a_2}{c_2}\right)^{1/2} - \frac{1}{4}\frac{c_1}{c_2}\right]^{-1/2} \quad \textbf{(Eq. S6)}$$

$$\xi = \left[\frac{1}{2}\left(\frac{a_2}{c_2}\right)^{1/2} + \frac{1}{4}\frac{c_1}{c_2}\right]^{-1/2} \quad \textbf{(Eq. S7)}$$

$$f_a = \frac{c_1}{\sqrt{4a_2c_2}} \quad \textbf{(Eq. S8)}$$

Scanning electron microscopy (SEM)

SEM micrographs were obtained using a field-emission NanoSEM 230 instrument with a 2 – 5 kV accelerating voltage and a secondary electron detector. EDX analysis was performed using the Nova NanoSEM 230, equipped with a silicon drift detector for EDX analysis (X-MAX Oxford Instruments), the accelerating voltage was set to 10 kV with a spot size of 5. The captured data is processed using the Aztec 6.0 software.

Transmission electron microscopy (TEM)

The samples were prepared using the following method: the materials were ground into a powder form, mixed with Milli-Q water at the concentration of 1 mg mL^{-1}. The resulting solution was ultrasonicated and then drop-cast onto 300 formvar coated carbon copper-mesh grids (ProSciTech, Australia). The size and morphology of particles were observed using a transmission electron microscope FEI Tecnai (FEI, OR, USA) at an accelerating voltage of 200 keV. Micrographs were recorded using an Eagle 2k CCD Camera (FEI) and digital micrograph.

X-ray photoelectron spectroscopy (XPS)

X-ray photoelectron spectroscopy (XPS) measurements were performed on a K-Alpha X-ray Photoelectron spectrometer system (Thermo Fisher Scientific, U.K.) with a monochromatic Al Kα source (energy 1480 eV) at 120 W over 500 μm at a 90° angle, background vacuum pressure was set at 2×10^{-9} mbar.

Thermogravimetric analysis (TGA)

The thermogravimetric analysis of 3D printed preceramic and ceramic materials were performed at a rate of 10 °C min^{-1} under nitrogen as well as under air using a TA Instrument TGA Q5000 thermogravimetric analyzer.

Differential scanning calorimetry (DSC)

Differential scanning calorimetry (DSC) to investigate the stabilization of PAN phase of 3D printed materials was performed at a rate of 5 °C min^{-1} under nitrogen using a TA Instrument DSC Q20.

Electrical conductivity measurement

For the electrical conductivity measurement, the 3D printed materials were thoroughly ground to obtain a fine powder. These powders were mixed with PTFE in 99:1 weight ratios and stretched and rolled with the help of ethanol. The as-obtained films (typical dimension: 1 cm × 1 cm × 0.05 cm) were air dried for 24 h and evaluated using a four-probe method (Keithley 2400 and LabTracer 2.9 software).

Raman spectroscopy

Analysis completed by Raman spectroscopy on Renishaw InVia Qontor Raman microscope. Instrument calibration was verified using internal silicon standard at 520 cm. Excitation was using 514 nm laser and 2400 mm/l grating. The samples were analyzed at 100% power for 5x 1s accumulations.

Synchrotron characterization

The *ex situ* synchrotron high-energy X-ray diffraction (HE-XRD) experiments were conducted at the beamline ID11 of the European Synchrotron (ESRF). HE-XRD data were collected using X-rays with an energy of ~87 keV (λ = 0.148 Å). The HE-XRD patterns were background corrected against an empty X-ray capillary, converted into reduced structure functions, S(q), and Fourier transformed into their corresponding atomic pair distribution functions (PDFs) using PDFgetX3.[2]

Viscosity measurements of resins

The measurements were performed using Brookfield Rheometer DV3T at 25 °C. Resin viscosity was measured at 20 rpm using the spindle CP-42.

Synthetic Procedures

RAFT polymerization of *n*-butyl acrylate in toluene

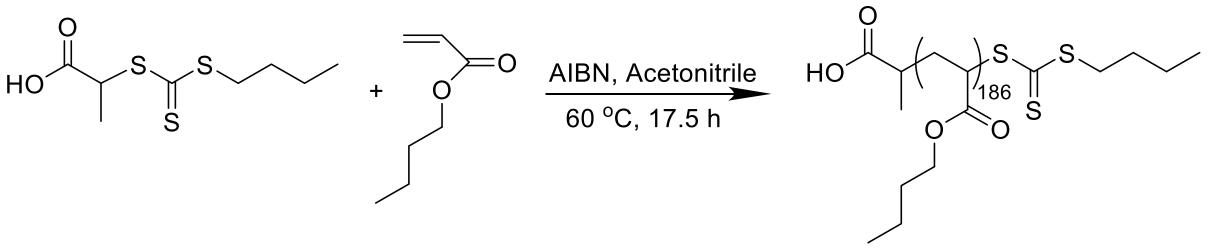

Fig. S1. Synthesis of PBA_{186}-CTA using RAFT polymerization of *n*-butyl acrylate.

Protocol for the synthesis of PBA_{186}-CTA: *n*-butyl acrylate (74.711 g, 0.583 mol), BTPA RAFT agent (0.695 g, 2.91×10^{-3} mol) and AIBN (0.2M solution in toluene, 2.186 mL, 4.37×10^{-4} mol) were dissolved in acetonitrile (147 mL). The mixture was deoxygenated by purging with nitrogen for 90 min, and then polymerized for 17.5 h at 60 °C. The reaction was stopped by cooling inside a freezer (-20 °C) for 30 min and exposing to air. The polymer solution was concentrated by rotary evaporation and used without further purification.

RAFT polymerization of acrylonitrile in dimethyl sulfoxide

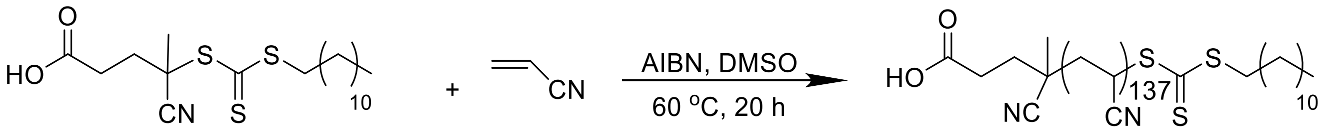

Fig. S2. Synthesis of PAN_{137} using RAFT polymerization of acrylonitrile.

Protocol for the synthesis of PAN_{137}: acrylonitrile (5 g, 0.094 mol), CDTPA RAFT agent (0.152 g, 3.77×10^{-4} mol) and AIBN (0.2M solution in toluene, 0.622 mL, 1.24×10^{-4} mol) were dissolved in DMSO (9.38 mL). The mixture was deoxygenated by purging with nitrogen for 30 min, and then polymerized for 20 h at 60 °C. The reaction was stopped by cooling inside a freezer (-20 °C) for 30 min and exposing to air. The polymer solution was concentrated by rotary evaporation and the polymer was recovered by precipitation into large excess of a mixture of methanol/water (1:1, v/v), filtered and then dried under vacuum at 23 °C.

Table S1. Characterization of synthesized polymers.

**Polymer**	**Conversion (%)**[a]	**M_n (theory) (g mol^{-1})**[b]	**SEC (RI)**[c]		**^{1}H NMR**[d]	
			**M_n (g mol^{-1})**	**$Đ$**	**X_n**	**M_n (g mol^{-1})**
PBA_{186}-CTA	93	24100	23850	1.09	186	24100
PAN_{137}	55	7700	27950	1.11	137	7700

[a] - Monomer conversion was calculated by ^{1}H NMR by comparing integrals of polymers and residual monomers (~ 5.8 – 6.5 ppm). [b] - M_n (theory) for PBA_{186}-CTA = ([BA]/[BTPA]) × conv. (BA) × MW(BA) + MW(BTPA). In a similar fashion, M_n (theory) for PAN_{137} was calculated. [c] - DMAc as eluent with polystyrene as calibration standards. [d] - ^{1}H NMR (400 MHz) at 298 K. The degree

of polymerization (X_n) for synthesized polymers was determined according to **Fig. S3**. M_n (NMR) for PBA_{186}-CTA = X_n(PBA-CTA) × MW(BA) + MW(BTPA). In a similar fashion, M_n (NMR) for PAN_{137} was calculated.

3D printing setup and procedure

A typical procedure for fabricating 3D printed objects is as follows: A 3D object was designed using Tinkercad 3D modelling software and the object was exported as an .stl file. The .stl file was opened using Photon Workshop where the Z lift speed was set to 3 mm/s and Z retract speed was set to 2 mm/s, while the Z lift distance was set to 6 mm. Printing parameters, such as layer thickness and exposure time, were defined in Photon workshop, sliced, and exported as .pws files for 3D printing. The .pws file copied to a flash drive for use with a LCD 3D printer (Anycubic Photon Mono SE) with a violet (λ_{max} = 405 nm) light LED array (I_0 = 2.0 mW cm^{-2}, as measured at the digital mask surface using a Newport 843-R power meter). For 3D printed PAN materials, the layer thickness was 50 μm, off time was 6 s, layer and bottom exposure times were 110 s, number of bottom layers was 2. Typical 3D printing resin formulations were prepared by combining the calculated amounts of PBA_{186}-CTA, AN, TMPTA, and TPO (**Table S2**). The resin was then added to the 3D printer vat, and the desired print program was run. After 3D printing was completed, the printed objects were separated from the build plate, washed with ethanol, air dried, and post-cured under violet light (λ_{max} = 405 nm) for 40 min. For 3D printed PAN/preceramic materials, the layer thickness was 100 μm, off time was 6 s, layer and bottom exposure times were 60 s, number of bottom layers was 2. After 3D printing was completed, the printed objects were separated from the build plate, washed with *n*-hexane, air dried, and post-cured under violet light (λ_{max} = 405 nm) for 40 min.

Pyrolysis of 3D printed PIMS PAN materials

Pyrolysis was carried out using a tubular furnace GSL-1700X (MTI Corporation). The 3D printed PAN materials were first stabilized under air flow at 220 °C at a heating rate of 1 °C min^{-1}. To investigate the effect of time on the stability of PAN domains, the materials were kept at 220 °C for 4, 8, or 12 h. Subsequently, the stabilized PAN materials were carbonized under N_2 flow with a heating rate of 1 °C min^{-1} and held at 600, 800, 1000, and 1200 °C for 1 h.

Additional Data

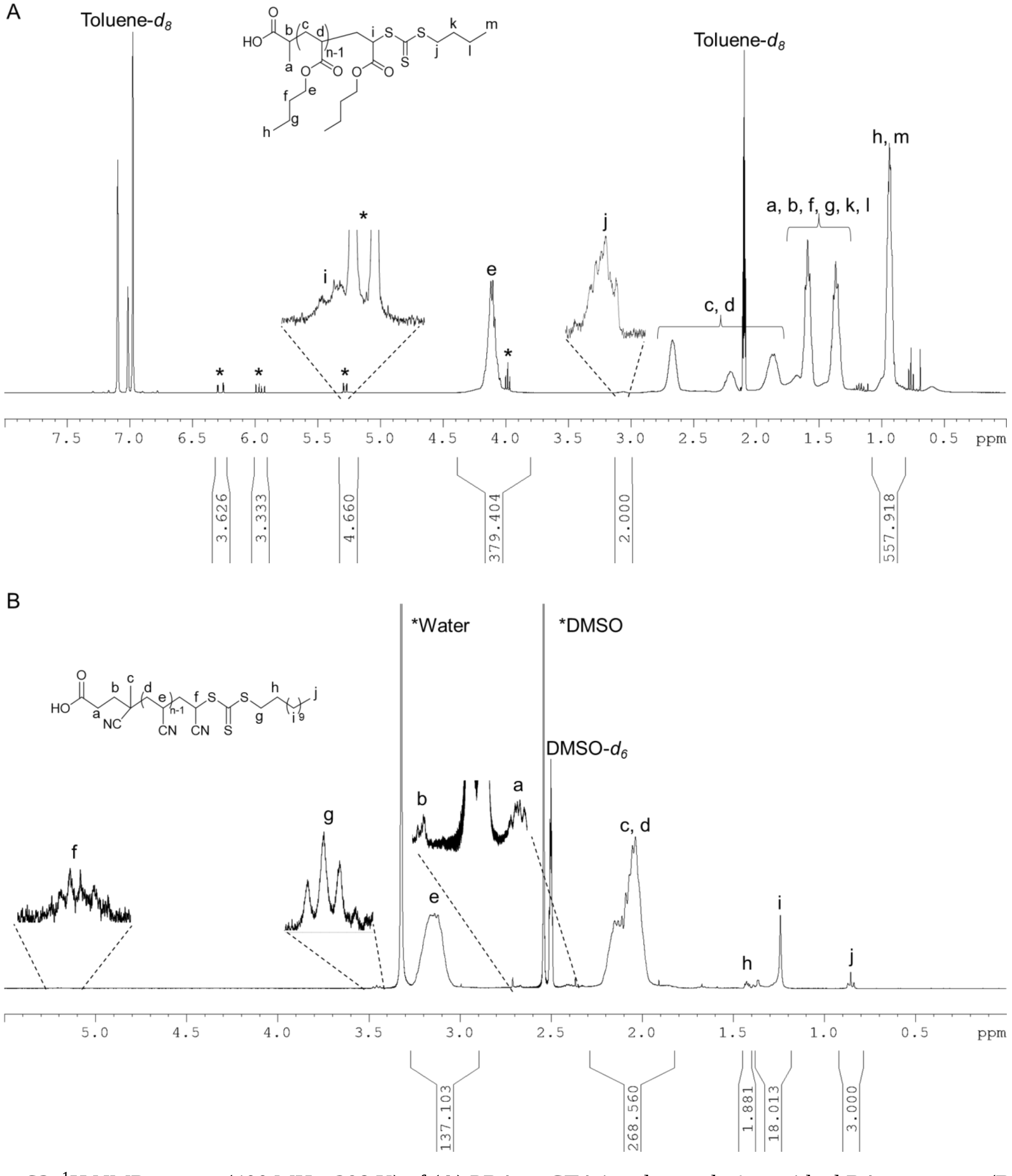

Fig. S3. ^{1}H NMR spectra (400 MHz, 298 K) of (**A**) PBA$_{186}$-CTA in toluene-d_8. * - residual BA monomer; (**B**) PAN$_{137}$ in DMSO-d_6.

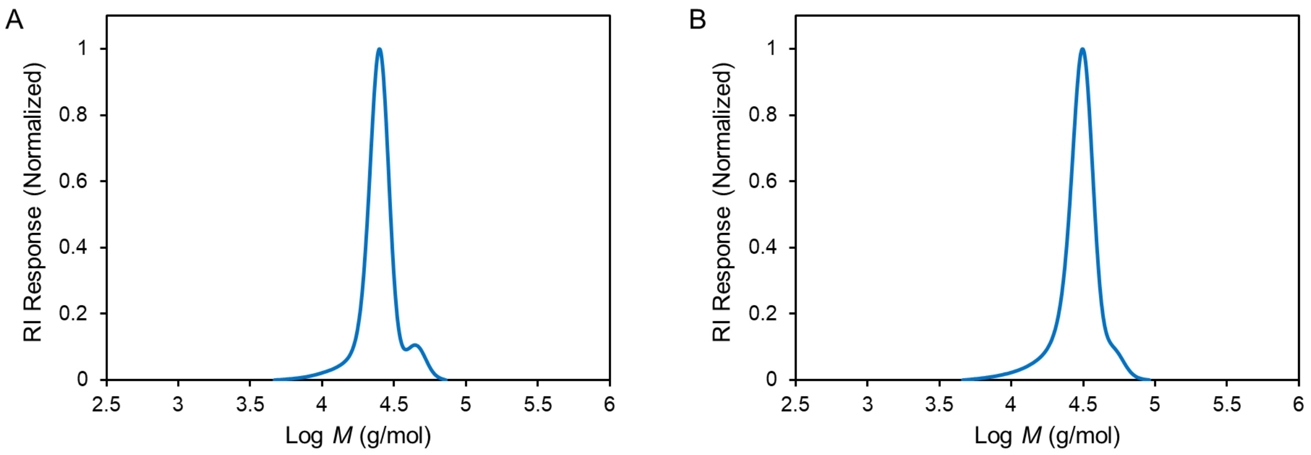

Fig. S4. SEC traces of (**A**) PBA_{186}-CTA and (**B**) PAN_{137}. Conditions: RI detector, DMAc as eluent, and PSTY calibration standards.

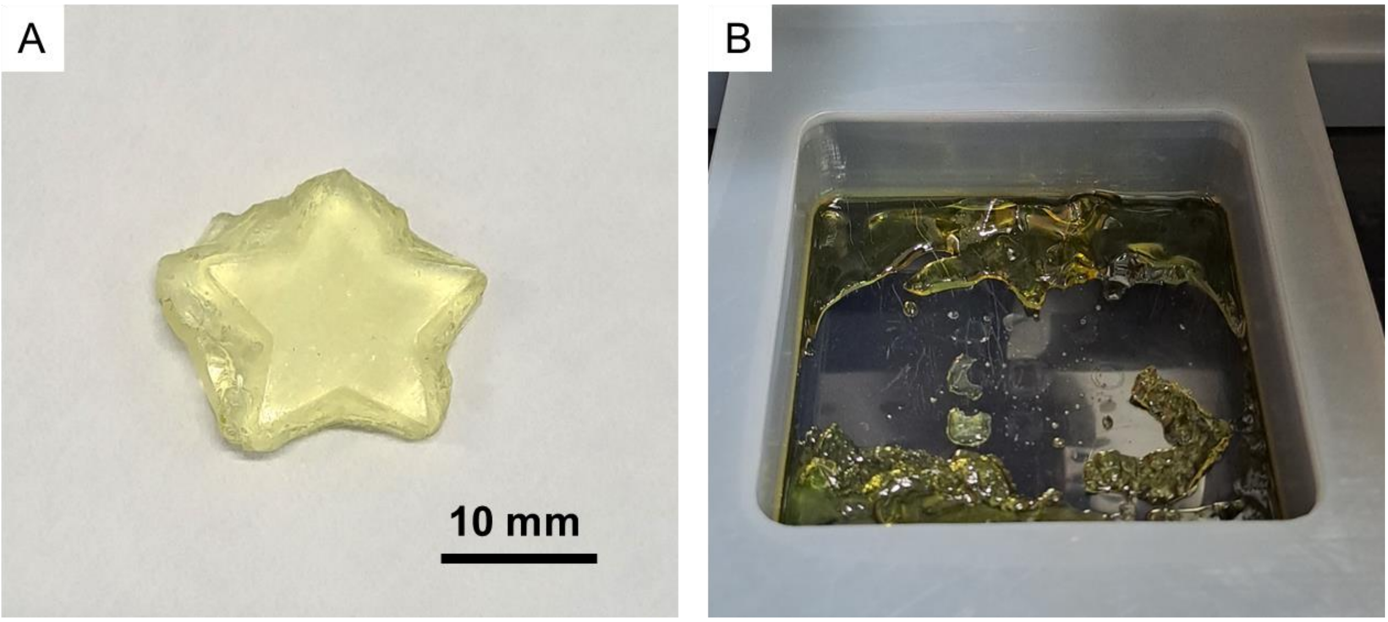

Fig. S5. A resin formulation without the addition of Sudan II resulted in the formation of (**A**) an overcured object and (**B**) gels inside the 3D printing vat.

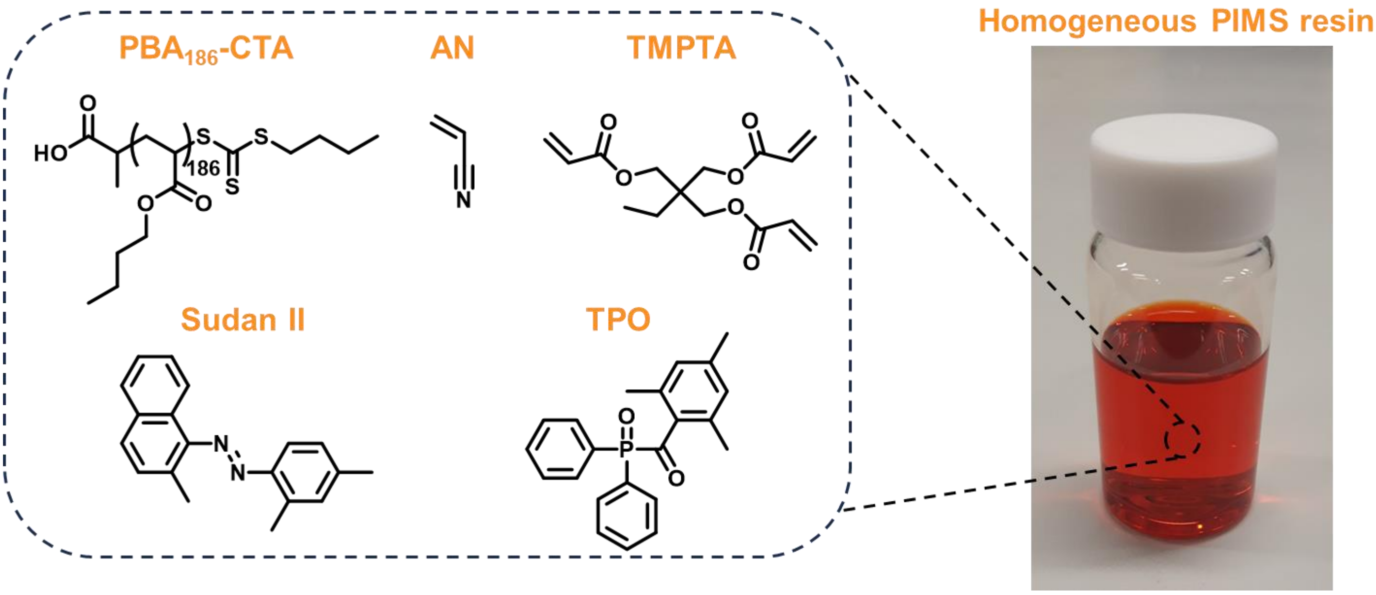

Fig. S6. The chemical structure of resin components and the photograph of a representative resin upon mixing all resin components.

<u>Note</u>: Upon mixing resin components, the transparent and homogeneous solution was formed. The viscosity of a representative resin formulation (Resin #2, **Table S2**) was 6.72 mPa s at 25 °C, remaining at a low level essential for DLP/LCD 3D printing.[3]

Table S2. Resin formulations for RAFT mediated PIMS 3D printing using PBA_{186}-CTA.

Resin #	Ratio	Resin components				
		**AN**	**TMPTA**	**PBA_{186}-CTA**	**TPO**	**Sudan II**
**1**	Molar	865	43.25	1	2.4	0.085
	wt%	54.9	15.3	28.8	1.0	0.03
**2**	Molar	970	24.25	1	2.4	0.085
	wt%	61.6	8.6	28.8	1.0	0.03
**3**	Molar	1045	10.45	1	2.4	0.085
	wt%	66.4	3.7	28.8	1.0	0.03
**4**	Molar	1095	2.19	1	2.4	0.085
	wt%	69.4	0.8	28.8	1.0	0.03

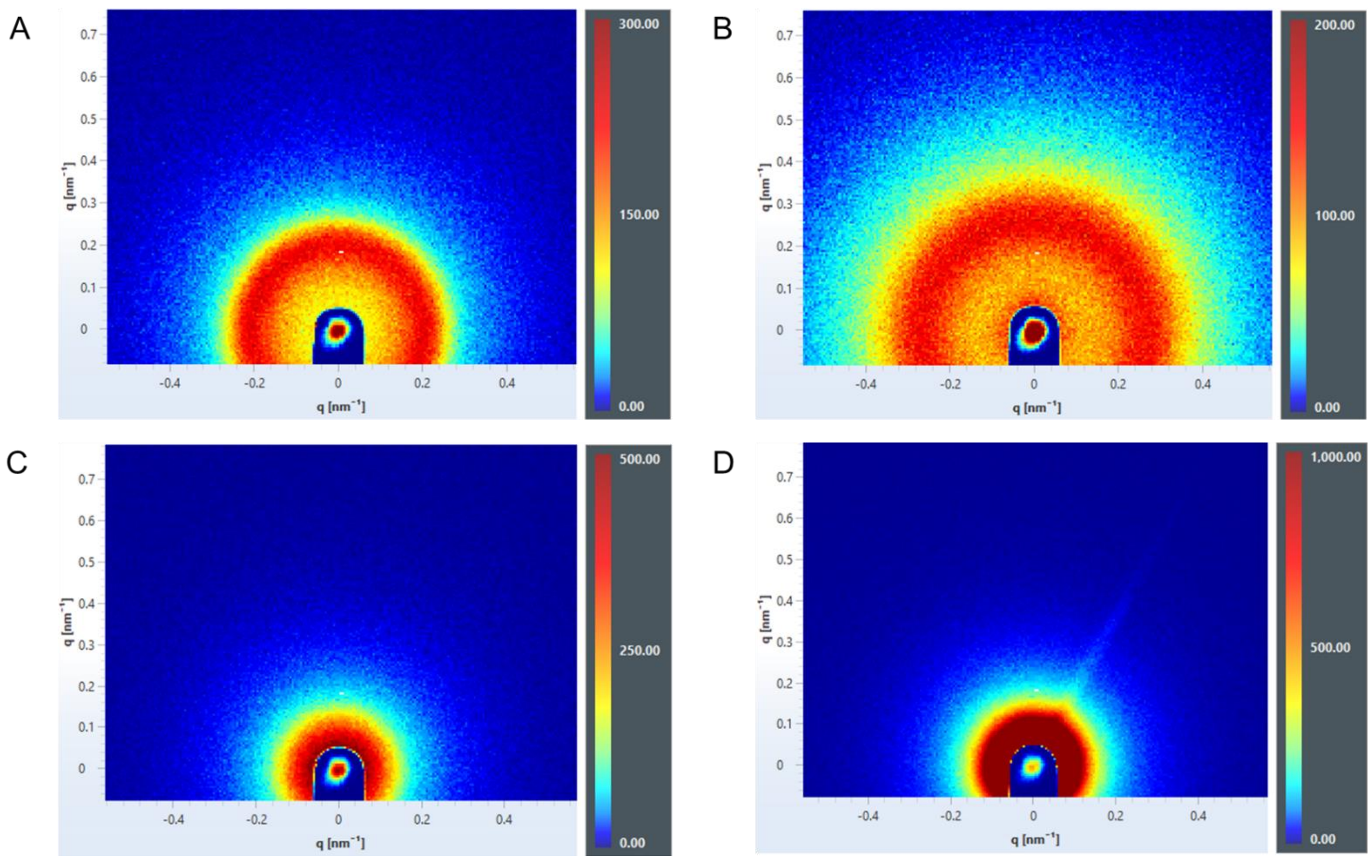

Fig. S7. 2D-SAXS profiles of PAN 3D printed materials with [AN]/[TMPTA] = (**A**) 20/1, (**B**) 40/1, (**C**) 100/1 and (**D**) 500/1 and 28.8 wt% of PBA_{186}-CTA loading.

<u>Note</u>: The intensity in the 2D patterns is the number of X-rays that were collected by that detector element (which is 75µm x 75µm) over the 5 minute collection time. It is not the same as the 1D patterns which are the fraction of scattered X-rays per cm of sample per steradian of detector.

Table S3. Parameter values obtained from fitting of SAXS peaks using the Teubner-Strey (T-S) model.

[AN]/[TMPTA][a]	a_2[b]	c_1[b]	c_2[b]	d_{SAXS} (nm)[c]	d_{TS} (nm)[d]	ξ (nm)[e]	ξ/d_{TS}[f]	f_a[g]
20	108	-3684	47979	31	30	15	0.49	-0.81
40	61	-966	6948	24	22	9	0.42	-0.74

[a] – the molar ratio of [AN]/[TMPTA]; [b] – parameters calculated from SAXS fitting using the T-S model; [c] – domain spacing determined from SAXS; [d] – domain spacing determined from T-S fitting using Equation S5; [e] – correlation length determined from T-S fitting using Equation S6; [f] – The ratio of ξ/d_{TS} is a measure of the domain size polydispersity, the smaller the ratio, the larger the polydispersity,[4] [g] – amphiphilicity factor determined using Equation S7.

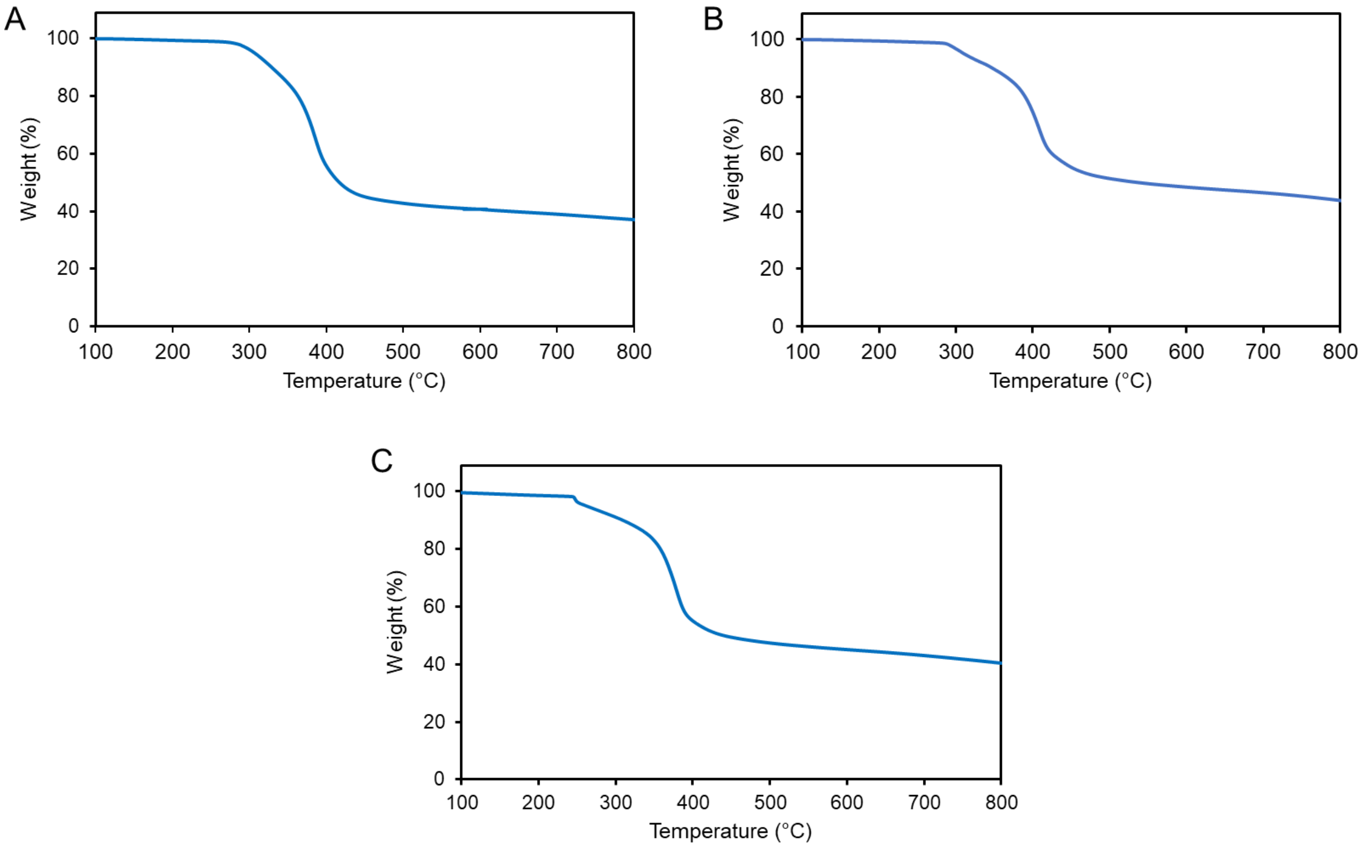

Fig. S8. TGA profiles of 3D printed PAN materials prepared with various molar ratio of [AN]/[TMPTA] of (**A**) 20/1, (**B**) 100/1, and (**C**) 500/1.

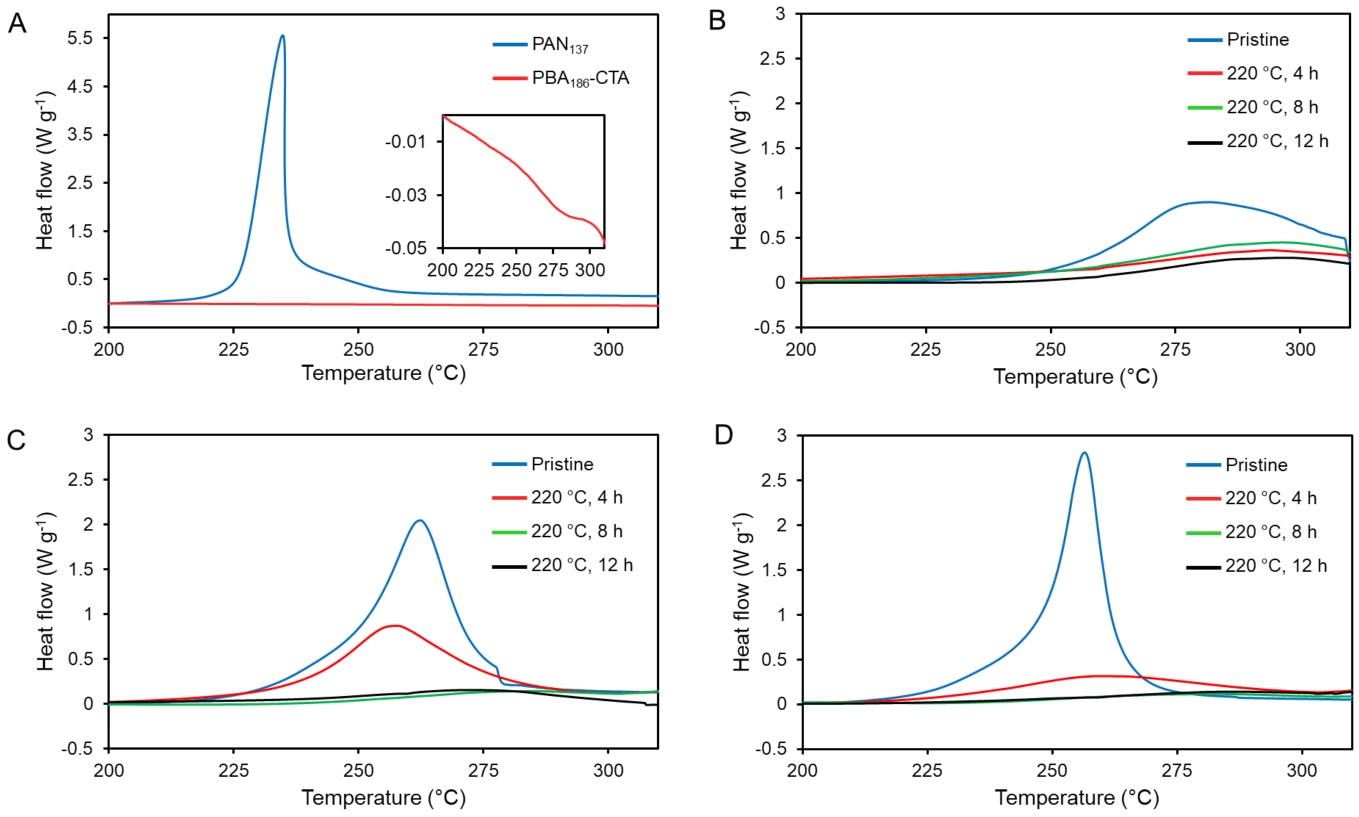

Fig. S9. DSC thermograms of (**A**) PAN_{137} and PBA_{186}-CTA (inset), and pristine and stabilized PAN materials 3D printed with various molar ratio of [AN]/[TMPTA] of (**B**) 20/1, (**C**) 100/1, and (**D**) 500/1. Samples were stabilized using the following protocol: 3D printed PAN materials were heated from room temperature to 220 °C at a rate of 1 °C min^{-1} under air and held at 220 °C for 4, 8, or 12 h in air. DSC heating rate: ramp 5 °C min^{-1} to 350 °C in nitrogen.

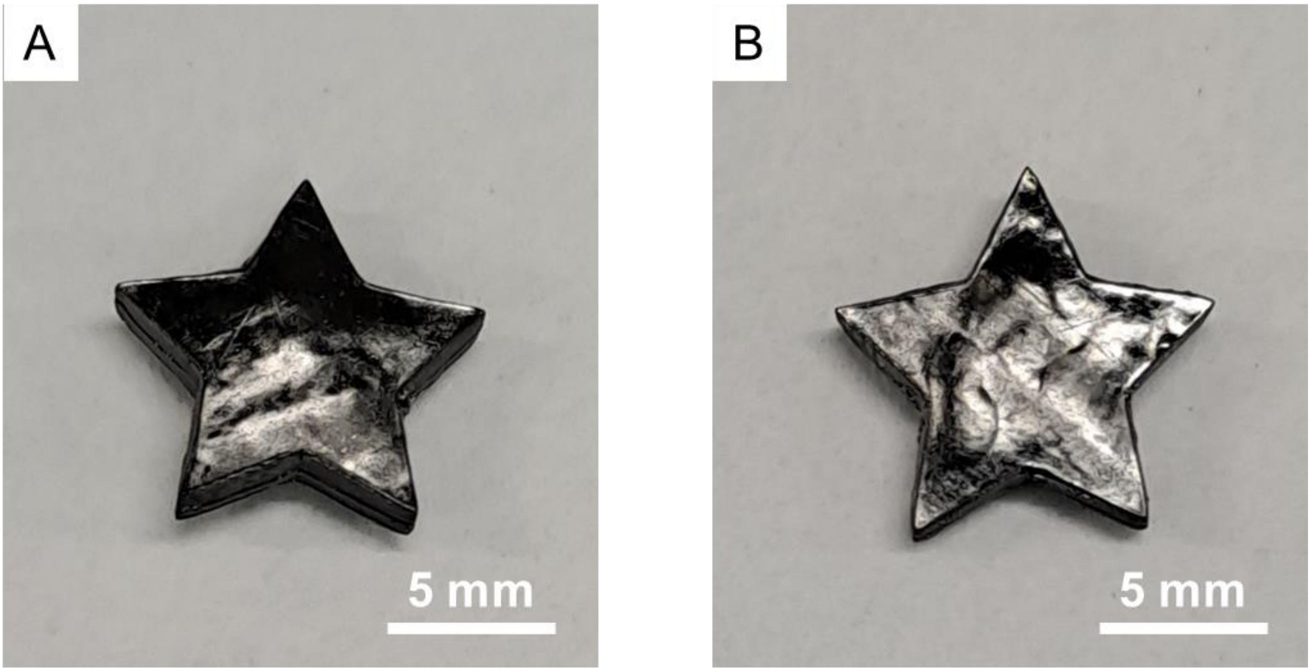

Fig. S10. Photographs of 3D printed objects carbonized at (**A**) 1000 and (**B**) 1200 °C. The materials were 3D printed using the molar ratio of [AN]/[TMPTA] = 40/1 and 28.8 wt% PBA_{186}-CTA.

<u>Note</u>: The carbonization at 1000 °C resulted in a shrinkage of the printed object by 32% with the obtained carbon yield of 42 wt%. In the case of carbonization at 1200 °C, the printed object shrunk by 34% and produced 39 wt% of carbon yield.

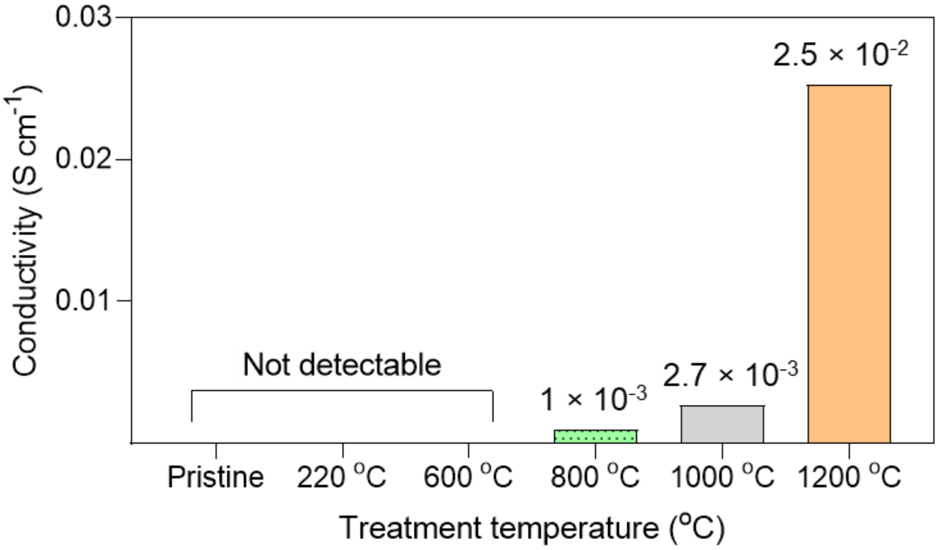

Fig. S11. Electrical conductivity of 3D printed PIMS PAN materials as a function of treatment temperature. The material was 3D printed using the molar ratio of [AN]/[TMPTA] = 40/1 and 28.8 wt% PBA_{186}-CTA.

Note: The pristine material and the materials treated at 220 and 600 °C did not exhibit detectable conductivity. Further increasing the carbonization temperature resulted in an overall increase in electronic conductivity. Specifically, as the carbonization temperature was raised from 800 to 1000 and further to 1200 °C, the conductivity values of 3D printed PAN materials increased from 1×10^{-3} to 2.7×10^{-3} and further to 2.5×10^{-2} S cm^{-1}.

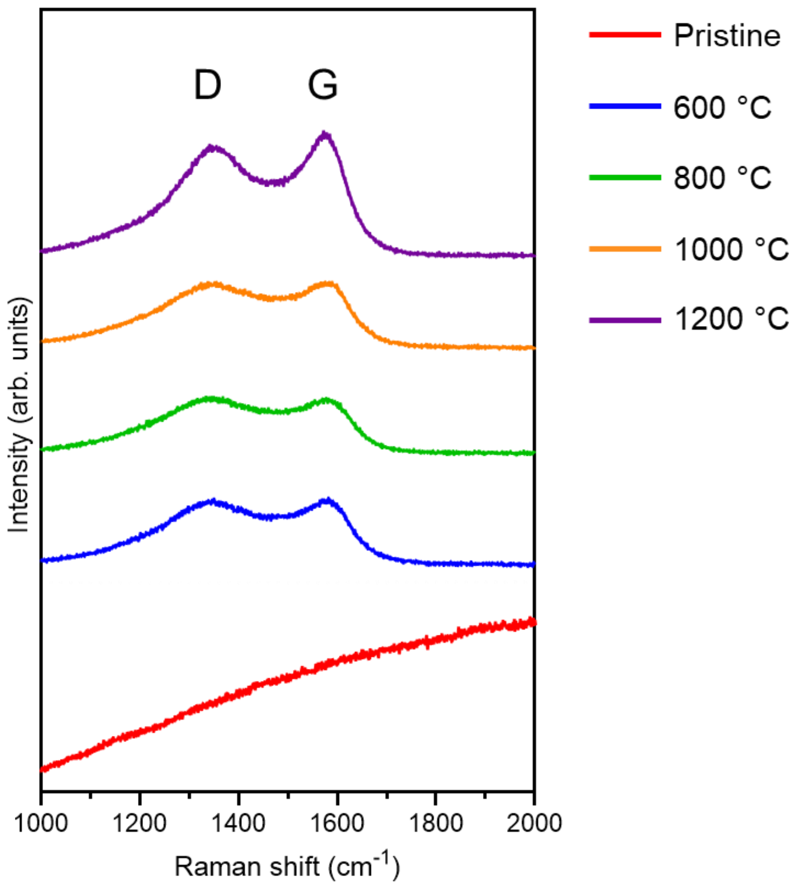

Fig. S12. Raman spectra of PIMS 3D printed PAN materials pyrolyzed at different temperatures. The materials were 3D printed using the molar ratio of [AN]/[TMPTA] = 40/1 and 28.8 wt% PBA_{186}-CTA. D – disordered band corresponds to sp^3 carbon species, G – graphitic band corresponds to graphitic species.

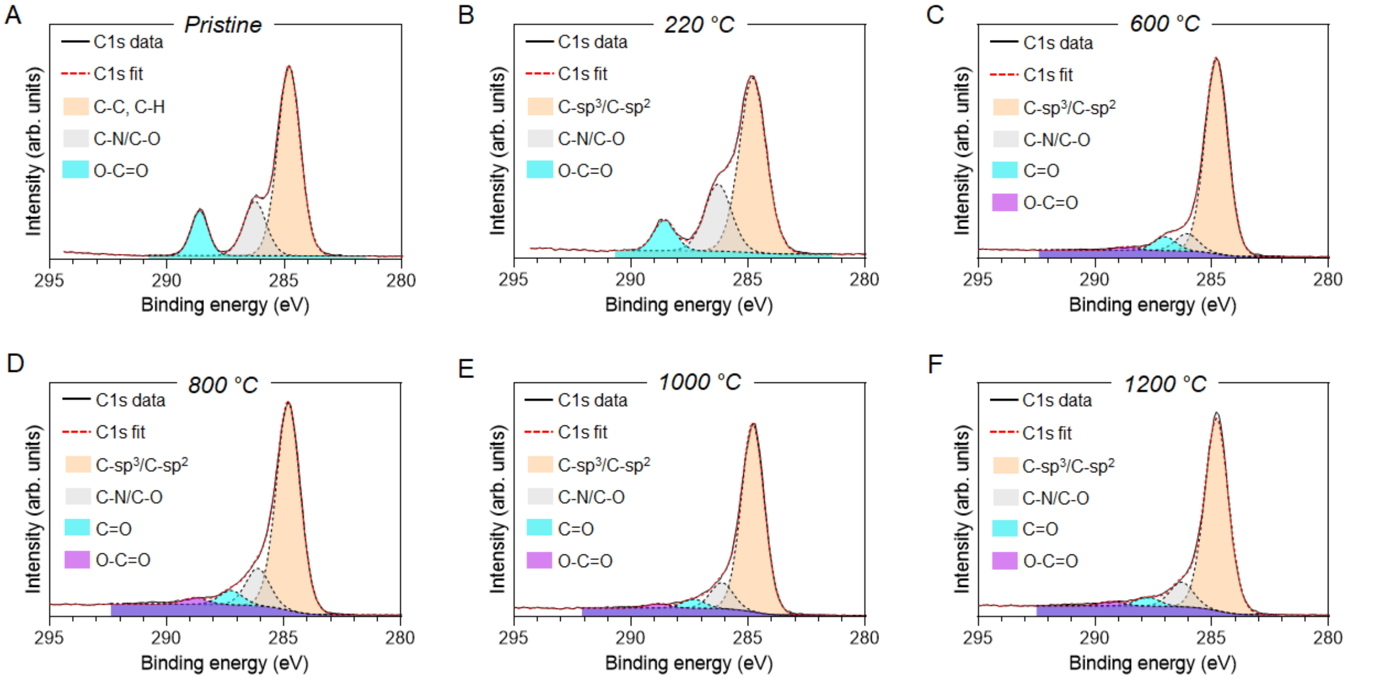

Fig. S13. Deconvoluted C1s XPS spectra of (**A**) pristine, treated at (**B**) 220, (**C**) 600, (**D**) 800, (**E**) 1000, and (**F**) 1200 °C 3D printed PIMS PAN materials.

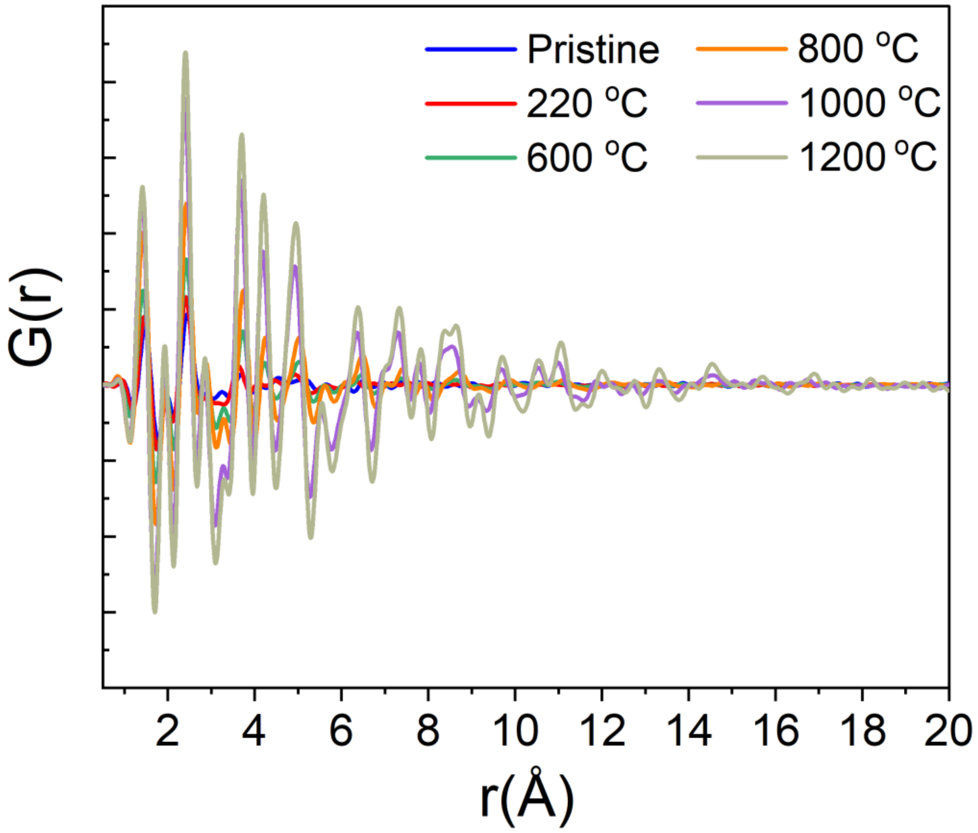

Fig. S14. Atomic PDFs of 3D printed PIMS PAN materials at different stages of *ex situ* pyrolysis, shown to 20 Å.

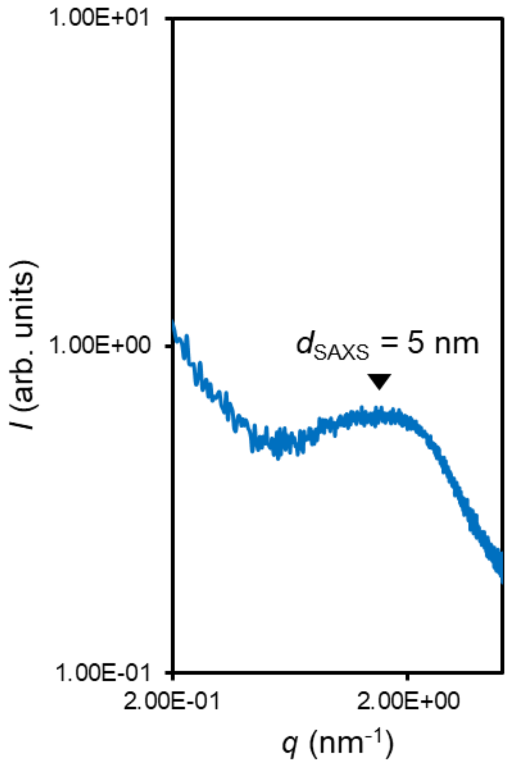

Fig. S15. Double logarithmic SAXS plot of the PIMS-PAN material after carbonization at 800 °C. The material was 3D printed using the molar ratio of [AN]/[TMPTA] = 40/1 and 28.8 wt% PBA_{186}-CTA. Protocol for stabilization stage: heating from ambient temperature to 220 °C at a heating rate of 1 °C min^{-1} in air and held at 220 °C for 1 h. Protocol for carbonization: heating from ambient temperature to 800 °C at a heating rate of 1 °C min^{-1} in nitrogen and held at 800 °C for 1 h.

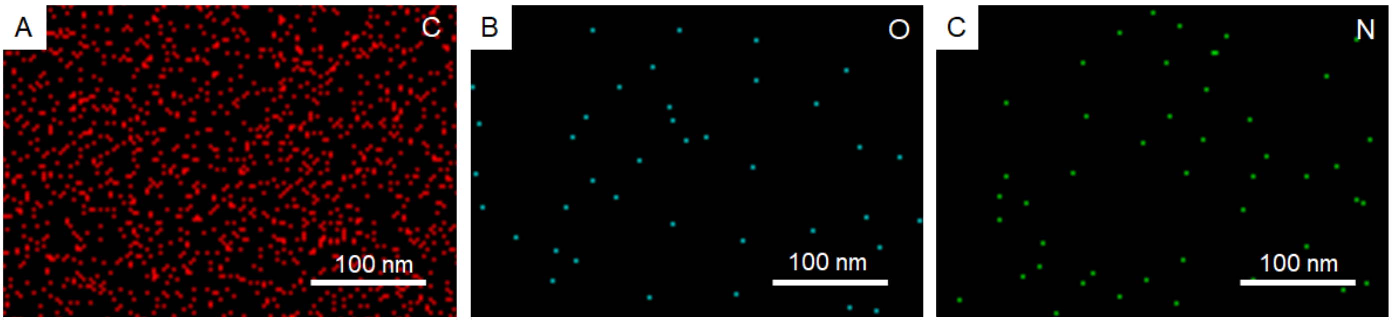

Fig. S16. Elemental mapping of (**A**) C, (**B**) O and (**C**) N of a carbonized object (800 °C, under nitrogen). The materials were 3D printed using the molar ratio of [AN]/[TMPTA] = 40/1 and 28.8 wt% PBA_{186}-CTA. Protocol for stabilization stage: heating from ambient temperature to 220 °C at a heating rate of 1 °C min^{-1} in air and held at 220 °C for 1 h. Protocol for carbonization: heating from ambient temperature to 800 °C at a heating rate of 1 °C min^{-1} in nitrogen and held at 800 °C for 1 h.

Table S4. The compositional analysis, binding energies and the corresponding chemical species of 3D printed nanostructured PAN-derived carbon materials.

Sample	Peak	Binding energy, eV	Chemical state	Atomic %
3D printed PAN-derived materials obtained upon pyrolysis at 800 °C	C1s	284.8	C-sp^3/C-sp^2	67.9
		286.1	C-N/C-O	12.3
		287.2	C=O	4.7
		288.8	O-C=O	2.2
	O1s	532.5	O=C	4.6
	Si2p	102.2	SiO_xC_y	0.6
	N1s	398.5	Pyridinic N	4.2
		400.5	Pyrrolic N	2.8
		401.7	Graphitic N	0.8

The materials were 3D printed using the molar ratio of [AN]/[TMPTA] = 40/1 and 28.8 wt% PBA_{186}-CTA. Protocol for stabilization stage: heating from ambient temperature to 220 °C at a heating rate of 1 °C min^{-1} in air and held at 220 °C for 1 h. Protocol for carbonization: heating from ambient temperature to 800 °C at a heating rate of 1 °C min^{-1} in nitrogen and held at 800 °C for 1 h.

References

[1] P. A. Small, *Journal of Applied Chemistry* **1953**, 3, 71.

[2] P. Juhas, T. Davis, C. L. Farrow, S. J. L. Billinge, *J. Appl. Crystallogr.* **2013**, 46, 560.

[3] K. Wang, W. Pan, Z. Liu, T. J. Wallin, G. van Dover, S. Li, E. P. Giannelis, Y. Menguc, R. F. Shepherd, *Adv. Mater.* **2020**, 32, 2001646.

[4] S. H. Chen, Chang, S. L., Strey, R. , *In Trends in Colloid and Interface Science IV; Zulauf, M., Lindner, P., Terech, P., Eds.; Dr. Dietrich Steinkopff Verlag* **1990**, 81, 30.
